# Spike burst-pause dynamics of Purkinje cells regulate sensorimotor adaptation

**DOI:** 10.1371/journal.pcbi.1006298

**Published:** 2019-03-12

**Authors:** Niceto R. Luque, Francisco Naveros, Richard R. Carrillo, Eduardo Ros, Angelo Arleo

**Affiliations:** 1 Sorbonne Université, INSERM, CNRS, Institut de la Vision, Paris, France; 2 Department of Computer Architecture and Technology, CITIC-University of Granada, Granada, Spain; Radboud Universiteit Nijmegen, NETHERLANDS

## Abstract

Cerebellar Purkinje cells mediate accurate eye movement coordination. However, it remains unclear how oculomotor adaptation depends on the interplay between the characteristic Purkinje cell response patterns, namely tonic, bursting, and spike pauses. Here, a spiking cerebellar model assesses the role of Purkinje cell firing patterns in vestibular ocular reflex (VOR) adaptation. The model captures the cerebellar microcircuit properties and it incorporates spike-based synaptic plasticity at multiple cerebellar sites. A detailed Purkinje cell model reproduces the three spike-firing patterns that are shown to regulate the cerebellar output. Our results suggest that pauses following Purkinje complex spikes (bursts) encode transient disinhibition of target medial vestibular nuclei, critically gating the vestibular signals conveyed by mossy fibres. This gating mechanism accounts for early and coarse VOR acquisition, prior to the late reflex consolidation. In addition, properly timed and sized Purkinje cell bursts allow the ratio between long-term depression and potentiation (LTD/LTP) to be finely shaped at mossy fibre-medial vestibular nuclei synapses, which optimises VOR consolidation. Tonic Purkinje cell firing maintains the consolidated VOR through time. Importantly, pauses are crucial to facilitate VOR phase-reversal learning, by reshaping previously learnt synaptic weight distributions. Altogether, these results predict that Purkinje spike burst-pause dynamics are instrumental to VOR learning and reversal adaptation.

## Author summary

Cerebellar Purkinje cells regulate accurate eye movement coordination. However, it remains unclear how cerebellar-dependent oculomotor adaptation depends on the interplay between Purkinje cell characteristic response patterns: tonic, high frequency bursting, and post-complex spike pauses. We explore the role of Purkinje spike burst-pause dynamics in VOR adaptation. A biophysical model of Purkinje cell is at the core of a spiking network model, which captures the cerebellar microcircuit properties and incorporates spike-based synaptic plasticity mechanisms at different cerebellar sites. We show that Purkinje spike burst-pause dynamics are critical for (1) gating the vestibular-motor response association during VOR acquisition; (2) mediating the LTD/LTP balance for VOR consolidation; (3) reshaping synaptic efficacy distributions for VOR phase-reversal adaptation; (4) explaining the reversal VOR gain discontinuities during sleeping.

## Introduction

**Fig 1:**
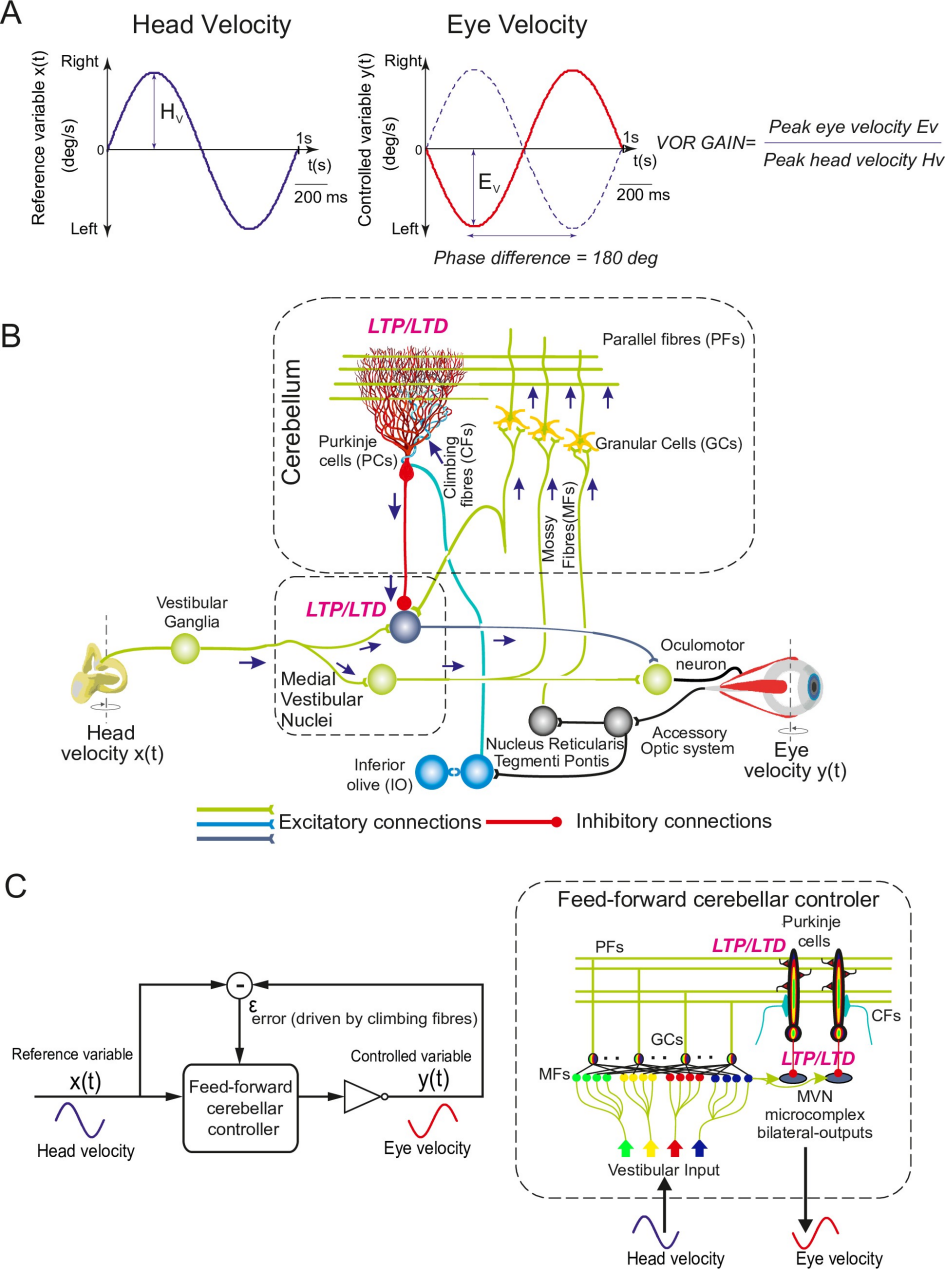
Vestibular ocular reflex (VOR) and cerebellar control loop. **(A)** Horizontal VOR (h-VOR) protocols compare head rotational movements (input) against the induced contralateral eye movements (output) via two measurements: the VOR gain, i.e. the ratio between eye and head speeds (E_v_ and H_v_, respectively); and the VOR phase, i.e. the temporal lag between eye and head velocity signals. **(B)** Schematic representation of the main neural layers, cells, connections, and plasticity sites considered in the cerebellar model. Mossy fibres (MFs) convey the sensory signals from the vestibular organ and they provide the input to the cerebellar network. MFs project sensorimotor information onto granular cells (GCs) and medial vestibular nuclei (MVN). GCs, in turn, project onto Purkinje cells through parallel fibres (PFs). Purkinje cells also receive excitatory inputs from the climbing fibres (CFs). CFs deliver the error signals encoding instructive terms that drive motor control learning. Purkinje cells integrate CF and PF inputs, thus transmitting the difference between head and eye movements. Finally, MVN are inhibited by Purkinje cells and provide the main cerebellar output. The cerebellar model implements different spike timing dependent plasticity mechanisms at multiple sites: PF-Purkinje cell, MF-MVN, and Purkinje cell-MVN synapses. **(C)** Cerebellar feed-forward control system comparing a known reference (head velocity or input variable) to the actual output (eye velocity) to quantify an error signal, whose delay matches the sensory-motor pathway delay (~100 ms) [7]. The cerebellum compensates for the difference between actual eye (represented as an inverter logic gate in this scheme) and head velocity profiles. The head velocity consists of a 1 Hz sinusoidal function iteratively presented to the cerebellar model, mimicking the sinusoidal frequency of the head rotation in experimental protocols [8].

The cerebellum controls fine motor coordination including online adjustments of eye movements [1]. Within the cerebellar cortex, the inhibitory projections of Purkinje cells to medial vestibular nuclei (MVN) mediate the acquisition of accurate oculomotor control [2, 3]. Here, we consider the role of cerebellar Purkinje cells in the adaptation of the vestibular ocular reflex (VOR), which generates rapid contralateral eye movements that maintain images in the fovea during head rotations (Fig 1A). The VOR is crucial to preserve clear vision (e.g., whilst reading) and maintain balance by stabilising gaze during head movements. The VOR is mediated by the three-neuron reflex arc comprised of connections from the vestibular organ via the medial vestibular nuclei (MVN) to the eye motor neurons[3–5]. VOR control is purely feed-forward [6] and it relies on several cerebellar-dependent adaptive mechanisms driven by sensory errors (Fig 1C). Because of its dependence upon cerebellar adaptation, VOR has become one of the most intensively used paradigms to assess cerebellar learning [6]. However, very few studies have focused on the relation between the characteristics spike response patterns of Purkinje cells and VOR adaptation, which is the main focus of this study.

Purkinje cells provide the major output of the cerebellum through MVN. Purkinje cells receive two main excitatory (glutamatergic) afferent currents (Fig 1B). The first excitatory input originates from the parallel fibres (PFs), i.e. the axons of the granule cells (GCs). The second comes from the climbing fibres (CFs), i.e. the projections of the inferior olive (IO) cells. These excitatory inputs drive Purkinje cell simple or complex spike patterns, respectively [9, 10]. Simple spikes of Purkinje cells are elicited topically at high frequencies [11, 12]. Complex spikes consist of a fast initial large-amplitude spike followed by a high-frequency burst [13]. This burst is made of several slower spikelets of smaller amplitude separated from one another by 2–3 ms [13–15]. Complex spikes are caused by the activation of a single IO neuron that produces a large electrical event in the soma of the post-synaptic Purkinje cell. This electrical event generates calcium-mediated action potentials in the Purkinje cell dendrites that, in turn, shape the complex spike. Simple spike activity is, in fact, mostly suppressed during complex spiking [15]. After each CF-evoked burst, a spike pause prevents Purkinje cells from firing for a period that increases in the presence of extra dendritic spikes [16–18]. The CF-evoked spike burst-pause sequences of Purkinje cell responses critically regulate the inhibitory (GABAergic) drive of MVN synapses, which determines the cerebellar output during sensorimotor adaptation. Therefore, understanding the dynamics of the characteristic Purkinje cell spike patterns is relevant to linking cerebellar cell properties to cerebellar-dependent behavioural adaptation. Recent studies have paved the road in gaining knowledge on the behavioural implication of Purkinje cell spike modes [3, 15, 19]. In particular, Herzfeld and colleagues suggested that the cerebellum predicts real-time motion of the eye through the organisation of Purkinje cells into clusters that share similar CF projections from the IO [3]. The combined activity of bursting and silent Purkinje cell populations can predict both the actual speed and direction of rapid accurate eye movements (saccades). However, these studies have not assessed the interplay between the different Purkinje cell spike patterns and the plasticity mechanisms at stake at MVN synapses in shaping sensorimotor adaptation. MVN neurons, in addition to receiving the inhibitory inputs from Purkinje cells, are also innervated by the excitatory afferents from the mossy fibres (MFs), which convey vestibular signals about head movements (Fig 1B). This vestibular information also converges onto Purkinje cells through the mossy fibre-granule cell-parallel fibre pathway (MF-GC-PF; Fig 1B). Therefore, the characteristic firing patterns of Purkinje cells are likely to play a key role in driving the associative plasticity mechanisms operating at MF-MVN excitatory synapses [20–22] and at Purkinje cells-MVN inhibitory synapses [23–26]. The CF-evoked spike burst-pause sequences of Purkinje cells depend indeed upon the activation of CFs, which are assumed to convey an ‘instructive’ signal encoding sensory error information [6, 15, 27]. Therefore, the properties of the CF-evoked spike burst-pause patterns (e.g., the relative duration of the bursts versus the pauses) reflect sensory error related information [15, 19, 28]. The activation of CFs is critical for inducing different forms of plasticity at PF-Purkinje cell synapses and, indirectly, at Purkinje cell-MVN synapses [29, 30]. Importantly, plasticity at MF-MVN synapses also seems to be dependent on Purkinje cell signals [31–33], generated through the MF-GC-PF pathway and through CF activation. Some computational studies have proposed that plasticity mechanisms at MF-MVN and Purkinje cell-MVN synapses as key factors in determining cerebellar adaptive gain control [31, 32, 34]. These models support the hypothesis of a two-state cerebellar adaptation process [35, 36], with a fast adaptive phase mediated by the cerebellar cortex (involving plasticity at Purkinje cell synapses) and a slow adaptive process occurring in deeper structures, involving plasticity at MVN synapses [33, 35–39]. However, these computational studies do not account for the interaction between the different spiking modes of Purkinje cells (in particular CF-evoked spike burst-pause dynamics) and the distributed plasticity mechanisms underpinning cerebellar adaptive control [34].

The spiking cerebellar model presented here addresses these issues within a VOR adaptation framework (Fig 1A and 1C). We simulate horizontal VOR (h-VOR) experiments with mice undertaking sinusoidal (~1 Hz) whole body rotations in the dark [40]. The model incorporates the main anatomo-functional properties of the cerebellar microcircuit, with synaptic plasticity mechanisms at multiple cerebellar sites (Fig 1B; see Materials & Methods).

## Results

### Spike burst–pause properties of model Purkinje cell responses

**Fig 2:**
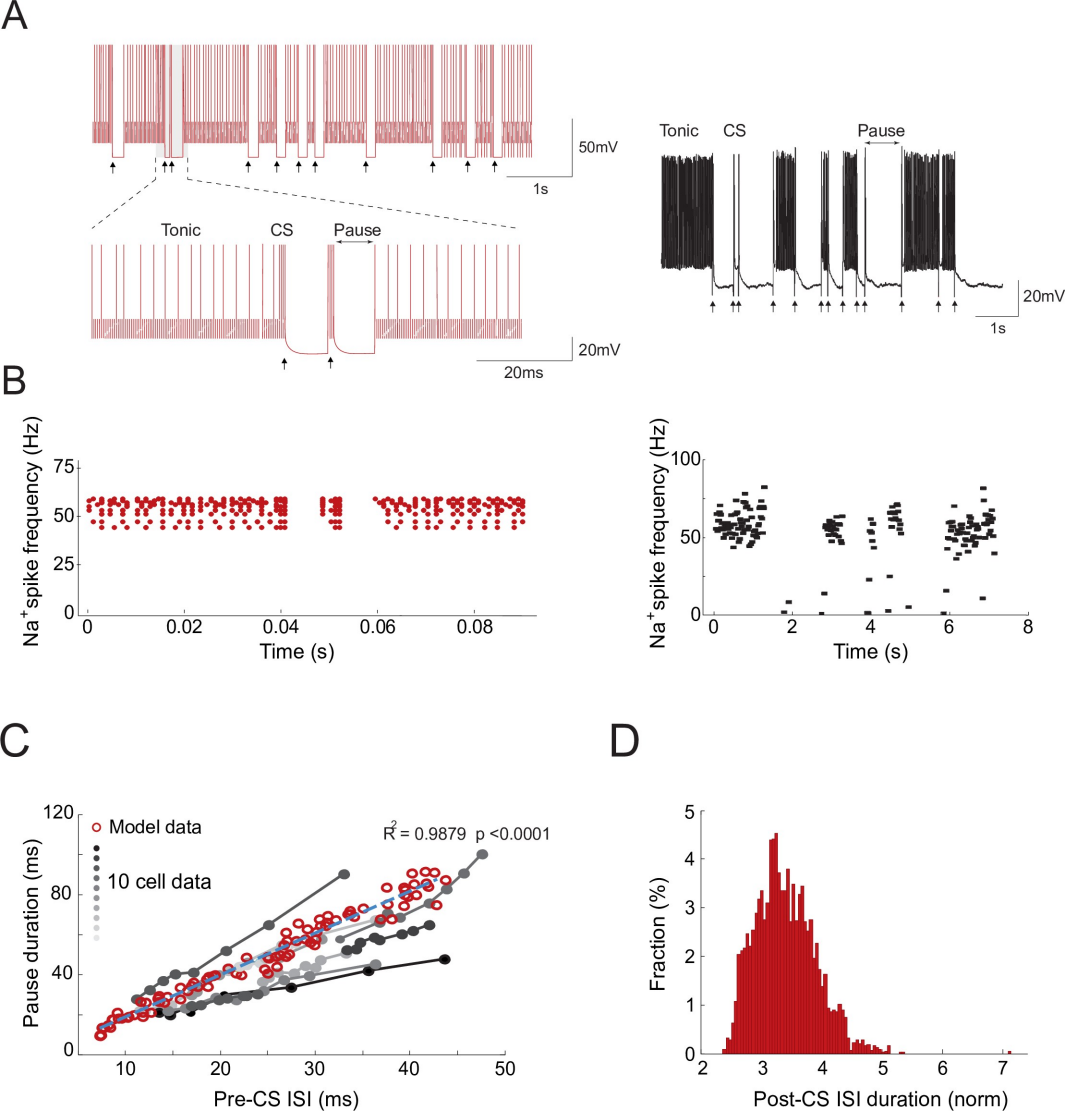
Spike burst–pause properties of model Purkinje cell responses. **(A)** Simulated (left) and electrophysiological (right) recordings of Purkinje cell spike outputs in response to CF spike excitatory postsynaptic potentials occurring at physiological frequencies (arrows) (data from [41]). CF discharges trigger transitions between Purkinje cell Na^+^ spike output and CF-evoked bursts and pauses via complex spikes. Here, the Purkinje cell model was run on the EDLUT simulator (see Methods). **(B)** Simulated (left) and experimental (right) Purkinje cell tonic spike frequency during CF discharges aligned with spike-grams in A (data from [41]). N = 10 Purkinje cells were simulated to compute the tonic spike frequency. **(C)** Relation between pause duration and pre-complex spike (pre–CS) inter spike intervals (ISIs) when increasing the amplitude of the injected current: model data (red circles, n = 1000) vs. experimental data [44] (grey to black dots). Grey-to-black lines represent individual cells (n = 10). The blue dashed line is the linear regression curve fitting model data. The model captures the relation between spike pause duration and pre-complex spike ISI duration observed electro physiologically [44]. **(D)** Distribution of ISI values following the complex spike (post-CS). The ISI duration is normalised to pre-CS ISI values. The Kurtosis for the distribution of post-CS ISI values is 4.24. The skewness is positive (0.6463), thus indicating an asymmetric post-CS ISI distribution. Kurtosis and skewness values were consistent with Purkinje cell data [44].

The detailed Purkinje cell model reproduces the characteristic response patterns observed experimentally: tonic simple spiking (20–200 Hz), complex spiking (bursts with high-frequency spikelet components up to 600 Hz), and post-complex spike pauses (Fig 2A). In the model, CF discharges trigger transitions between the Purkinje cell Na^+^ spike output, CF-evoked bursts, and post-complex spike pauses. As evidenced in [41], in *in-vitro* slice preparations at normal physiological conditions, 70% of Purkinje cells spontaneously express a trimodal oscillation: a Na^+^ tonic spike phase, a Ca-Na^+^ bursting phase, and a hyperpolarised quiescent phase. On the other hand, Purkinje cells also show spontaneous firing consisting of a tonic Na^+^ spike output without Ca- Na^+^ bursts [41–43]. McKay et al. [41] report Purkinje cell recordings exhibiting a tonic Na^+^ phase sequence followed by CF-evoked bursts (via complex spikes) and the subsequent pause (Fig 2A). The frequency of Purkinje cell Na^+^ spike output decreases with no correlation with the intervals between CF discharges [41]. The model mimics this behaviour under similar CF discharge conditions (Fig 2B). It also replicates the relation between spike pause duration and *pre-*complex spike inter-spike interval (ISI) duration observed through electrophysiological recordings [44] (Fig 2C; R^2^ = 0.9879; p<0.0001). Only ISIs immediately following complex spikes were considered for this analysis. This relation was measured by maintaining the CF stimulation constant whilst incrementally increasing the amplitude of the PF input current. The probability distribution of *post*-complex spike ISIs is also consistent with experimental data [44] (Fig 2D). The kurtosis (‘peakedness’) of the ISI distribution is 4.24, which is in the range of kurtosis values measured after tetanisation of mouse Purkinje cells [44]. Model *post*-complex spike ISI values are skewed rightward (positive skewness value of 0.6463), consistently with the asymmetric distribution shape observed experimentally [44]. Finally, the duration of the model post-complex spike pauses is non-linearly related to burst duration (S1A and S1B Fig), assuming that CF stimuli carrying large error-related signals (as during VOR adaption) elicit both somatic and extra dendritic Purkinje spikes [16–18].

### Role of cerebellar Purkinje spike burst-pause dynamics in VOR adaptation

**Fig 3:**
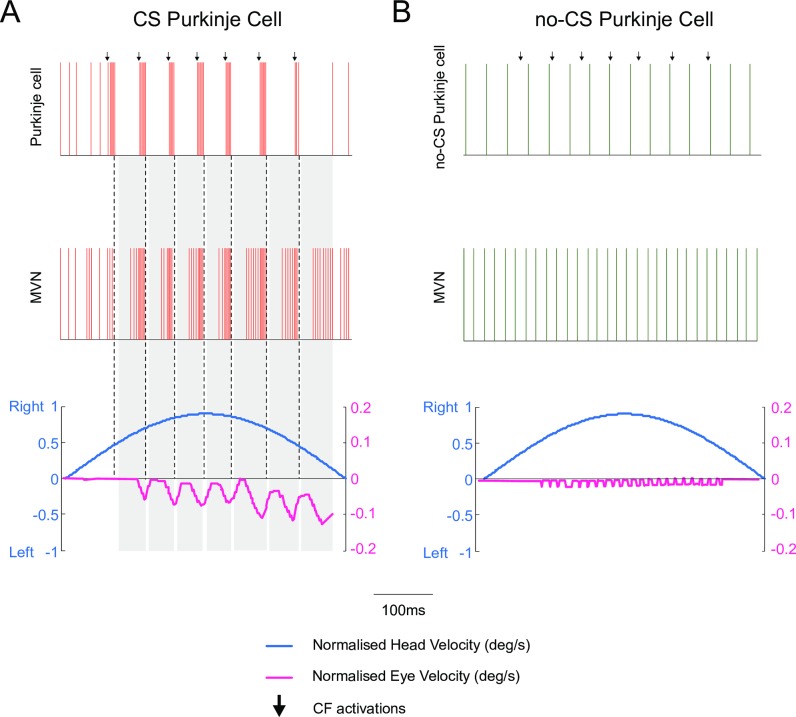
Purkinje post–complex spike pauses act as a gating mechanism for early coarse VOR in the absence of cerebellar adaptation. Only half of h-VOR cycle is represented. Two equal cerebellar network configurations except for the Purkinje cell dynamics were compared under equal stimulation. **(A)** The first model accounts for CF-evoked Purkinje spike burst-pause dynamics. CF stimulation generates complex spikes and subsequent post–complex spike pauses. The latter allows MFs to drive directly the immediate activation of MVN, which facilitates an early but rough eye movement compensation for head velocity. **(B)** The second model only exhibits Purkinje tonic firing (i.e., complex spiking is blocked through the blockade of muscarinic voltage-dependent channels, see Methods), which prevents MFs from eliciting any baseline MVN compensatory output. See S2 and S3 Figs for a sensitivity analysis of parameters regulating the LTD/LTP balance at PF-Purkinje cell and MF-MVN synapses. See also S4 Fig for the same parameter sensitivity analysis in the absence of Purkinje spike burst-pause dynamics.

We assessed h-VOR adaptation by simulating a 1 Hz horizontal head rotation to be compensated by contralateral eye movements (Fig 1A). First, we tested the role of Purkinje spike burst-pause dynamics in the absence of cerebellar learning, i.e. by blocking synaptic plasticity across all model projections (i.e., MF-MVN, PF-Purkinje cell, Purkinje cell-MVN). Synaptic weights were initialised randomly and equally within each projection set. The CF input driving Purkinje cells was taken as to signal large retina slips, which generated sequences of complex spikes made of 4 to 6 burst spikelets [15] (Fig 3A, top). The elicited Purkinje spike burst-pause sequences shaped the temporal disinhibition of target MVN neurons, allowing the incoming input from MFs to drive MVN responses (Fig 3A, middle). This facilitated a coarse baseline eye motion (Fig 3A, bottom). Blocking complex spiking in the Purkinje cell model (through the blockade of muscarinic voltage-dependent channels, see Methods) prevented MF activity from eliciting any baseline MVN compensatory output (Fig 3B). These results suggest that the gating mechanism mediated by Purkinje spike burst-pause sequences, which encode transient disinhibition of MVN neurons, is useful for early and coarse VOR, prior to the adaptive consolidation of the reflex through cerebellar learning.

We then activated the LTD/LTP plasticity mechanisms at MF-MVN, PF-Purkinje cell, and Purkinje cell-MVN synapses (see Materials & Methods). During 10000 s, the model faced a 1 Hz horizontal head rotation, and cerebellar h-VOR learning took place to generate compensatory contralateral eye movements. A sensitivity analysis identified the critical LTD/LTP balance at MF-MVN and PF-Purkinje cell synapses in order to achieve VOR adaptation (in terms of both gain and phase). This analysis predicts a very narrow range of values for which LTP slightly exceeding LTD at MF-MVN synapses ensures learning stability through time. By contrast, PF-Purkinje cell synapses admitted a significantly broader range for the LTD/LTP ratio (S2 and S3 Figs). The same parameter sensitivity analysis for the cerebellar model with no bursting and pause dynamics shows a much wider range of values for the LTD/LTP balance at both PF-Purkinje cell and MF-MVN synapses (S4 Fig).

**Fig 4:**
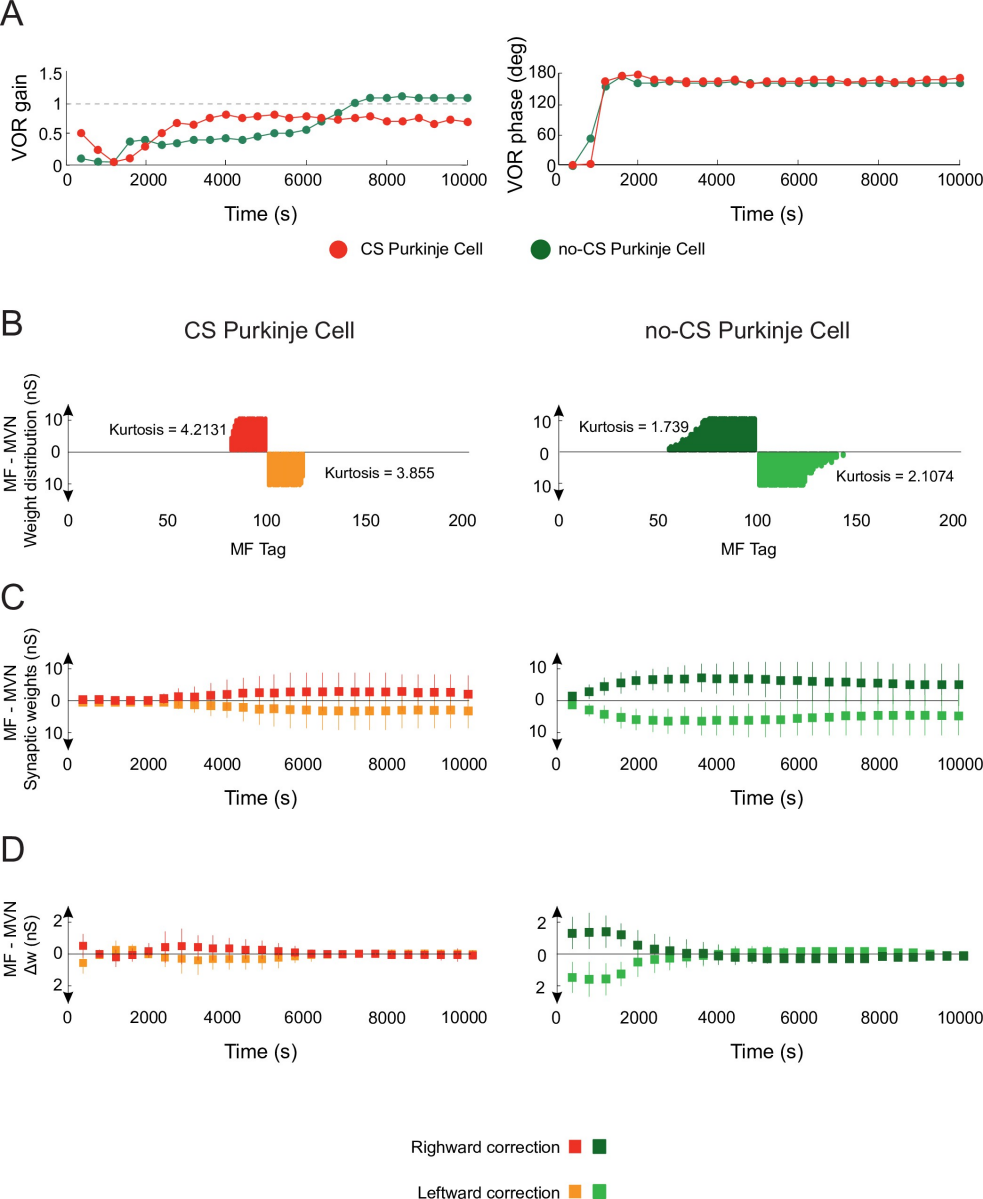
Role of Purkinje spike burst-pause dynamics in VOR cerebellar adaptation. **(A)** VOR gain and phase adaptation with (purple curve) and without (green curve) CF-evoked Purkinje spike burst-pause dynamics. VOR cerebellar adaptation starts with zero gain owing to the initial synaptic weights at PF and MVN afferents (Table 5). Purkinje spike burst-pause dynamics provides better VOR gain adaptation (in terms of both rate and precision) converging to gain values within [0.8–0.9] (S5 Fig), which are consistent with experimental data [40, 45, 46]. **(B)** Purkinje complex spiking allows a sparser weight distribution (with higher Kurtosis) to be learnt at MF-MVN synapses, with significantly lesser MF afferents needed for learning consolidation. **(C)** The model endowed with Purkinje complex spiking updates less MF afferents during learning consolidation but their synaptic range is fully exploited. **(D)** The averaged synaptic weight variations are more selective during the adaptive process in the presence of Purkinje spike burst-pause dynamics, yet the standard deviation remains equal.

A comparison of VOR adaptation accuracy in the presence vs. absence of CF-evoked Purkinje spike burst-pause dynamics shows that VOR gain plateaued three times faster in the presence of Purkinje complex spikes (Fig 4A, left). Also, the VOR gain converged to [0.8–0.9], which is consistent with experimental recordings in mice [40], monkeys [45], and humans [46] (S5 Fig). Conversely, without Purkinje bursting-pause dynamics the VOR gain saturated to a value >1 (i.e. over learning) at the end of the adaptation process. In terms of VOR phase, convergence to 180° (i.e., well synchronised counter-phase eye movements) was reached after approximately 1000 s under both conditions (Fig 4A, right).

A more accurate VOR gain adaptation in the presence of Purkinje complex spiking reflected a more selective synaptic modulation across learning (Fig 4B–4D). In particular, Purkinje spike burst-pause dynamics facilitated a sparser weight distribution at MF-MVN synapses (Fig 4B), which ultimately shaped VOR adaptation [21]. Indeed, Purkinje burst sizes, which were assumed to reflect the sensed errors [15, 19, 28], regulated the inhibitory action of Purkinje cells on MVN, and induced error-dependent LTD at MF-MVN synapses (see Materials & Methods). On the other hand, post-complex spike pauses (disinhibiting MVN) induced error-dependent LTP at MF-MVN synapses (the larger the error, the larger the burst size, and the wider the post-complex spike pause in the presence of extradendritic Purkinje cell spikes, S1 Fig. At the beginning of VOR adaptation, the error was larger, and so were the burst and pause durations. Because the durations of pauses remained always larger than bursts (S1 Fig. LTP dominated over LTD at MF-MVN synapses, increasing the learning rate. Therefore, the spike burst-pause dynamics enhanced the precision of cerebellar adaptation at MVN cells, by *(i)* recruiting the strictly necessary MF-MVN projections (i.e., higher kurtosis value of the synaptic weight distribution; Fig 4B), *(ii)* making a better use of the synaptic range of selected projections (larger standard deviations with lower overall gains; Fig 4C), and the rate by *(iii)* varying synaptic weights selectively (lower averaged synaptic weight variations; Fig 4D).

### Purkinje spike burst-pause dynamics facilitates VOR phase-reversal learning

**Fig 5:**
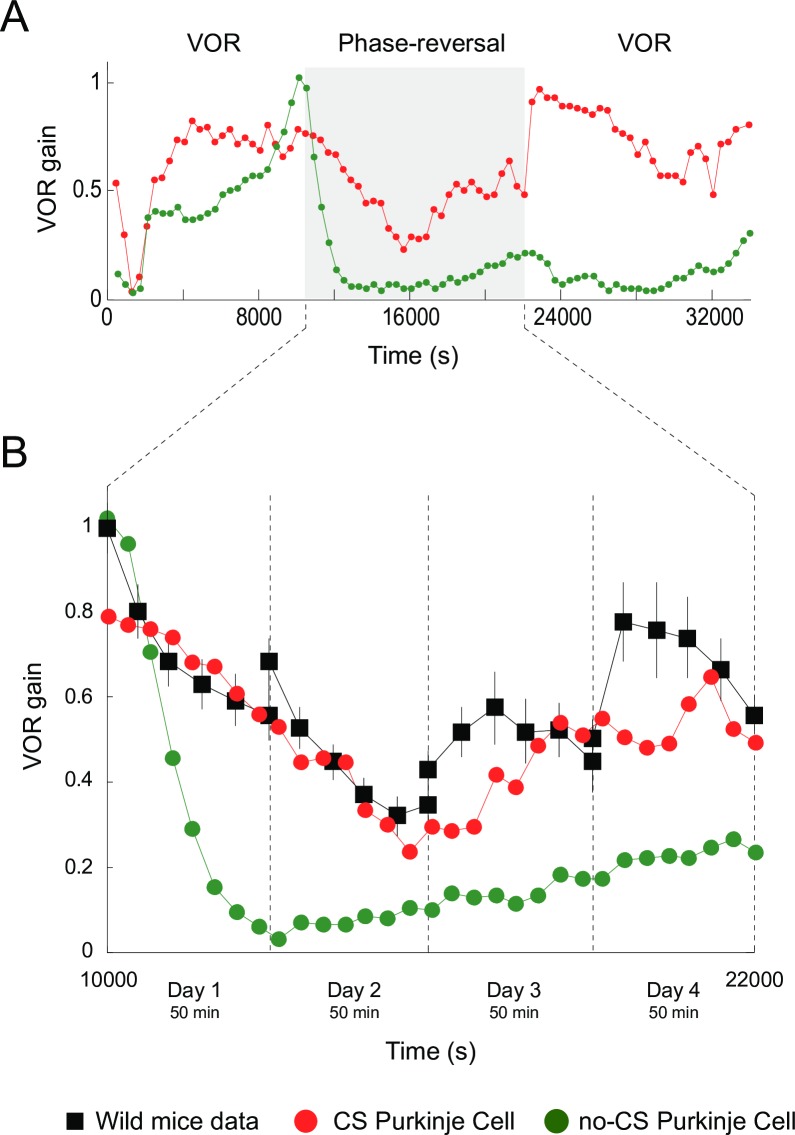
Purkinje spike burst-pause dynamics facilitates VOR phase-reversal learning. **(A)** VOR gain adaptation with (red curve) and without (green curve) Purkinje spike burst-pause dynamics during: VOR adaptation (first 10000 s), phase-reversal learning (subsequent 12000 s), and normal VOR restoration (remaining 12000 s). **(B)** Purkinje spike burst-pause dynamics provides fast learning reversibility, consistently with experimental recordings [2]. By contrast, phase-reversal VOR learning is impaired in the absence of Purkinje complex spiking. See S6 Fig for the time course of VOR phase-reversal learning.

Phase-reversal VOR is induced when a visual stimulus is given simultaneously in phase to the vestibular stimulation but at greater amplitude (10% more) [29]. This creates a mismatch between visual and vestibular stimulation making retinal slips reverse direction[47]. Cerebellar learning is deeply affected by VOR phase reversal since the synaptic weight distribution at both PF-Purkinje cell and MF-MVN synapses must be reversed. Here, we first simulated an h-VOR adaptation protocol (1 Hz) during 10000 s (as before). Then, h-VOR phase reversal took place during the next 12000 s. Finally, the normal h-VOR had to be restored during the last 12000 s (Fig 5). Our results suggest that the presence of Purkinje spike burst-pause dynamics is instrumental to phase-reversal VOR gain adaptation (Figs 5A and S7) allowing for fast VOR learning reversibility consistently with experimental recordings [2] (Fig 5B). Conversely, the absence of Purkinje complex spiking led to impaired VOR phase-reversal learning with significant interference (Fig 5A and 5B). The two models (i.e., with and without Purkinje complex spiking) behaved similarly in terms of VOR phase adaptation during the same reversal learning protocol (S6 Fig).

**Fig 6:**
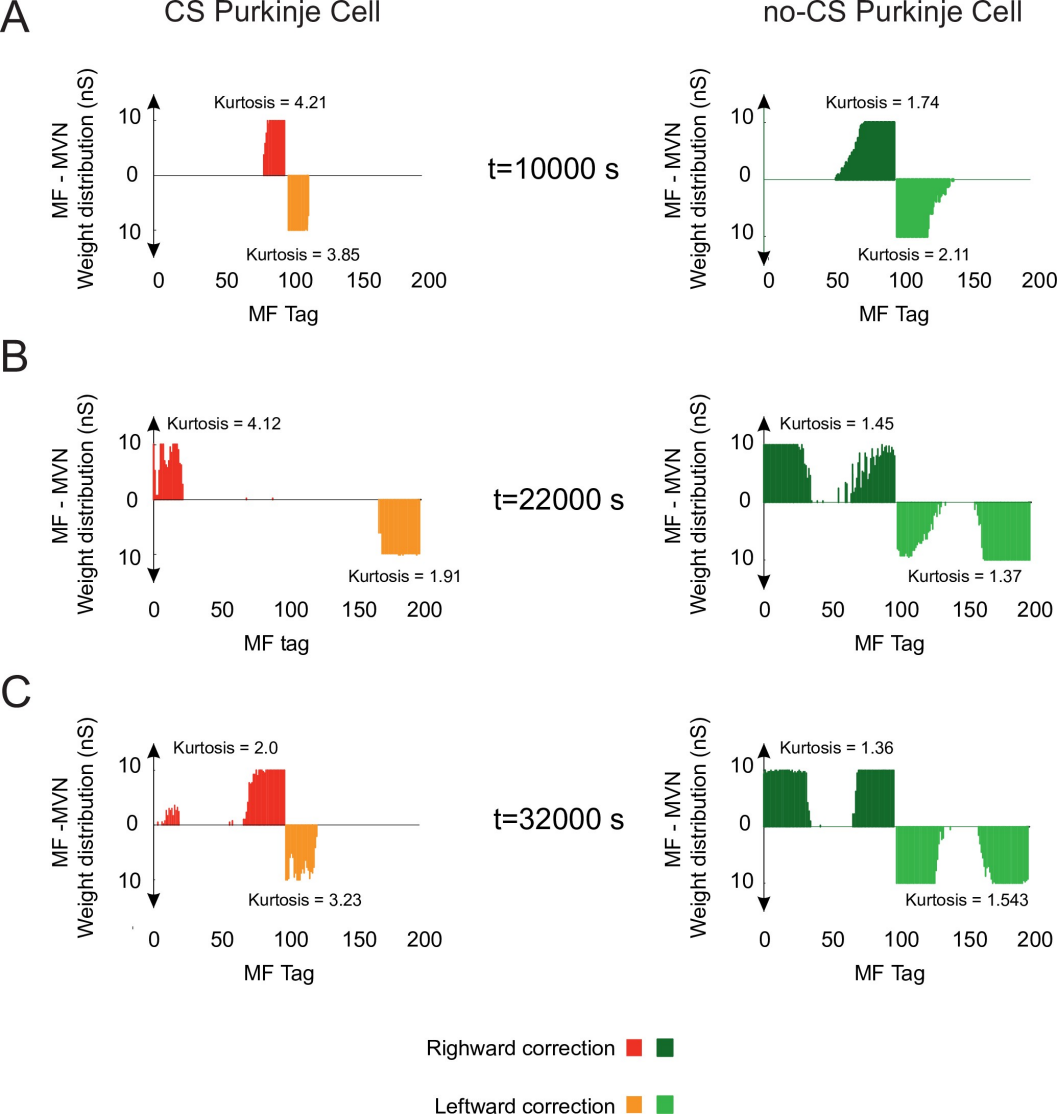
Evolution of synaptic weight distributions during VOR phase-reversal learning. **(A**) Only the sparser and more selective distribution of MF-MVN synaptic weights resulting from the interplay between bursts and post-complex spike pauses facilitates an efficient reshaping of the learnt patterns (**B**), allowing phase-reversal learning to be achieved (**C**).

VOR phase-reversal learning demanded first the reduction of the VOR gain, which can be regarded as a ‘forgetting phase’ (Fig 5B, days 1&2). Then, a ‘synchronisation phase’ took place with a reverse adaptive action that gradually increased the VOR gain (Fig 5B, days 3&4). During the forgetting phase, LTD dominated over LTP at MF-MVN synapses (Purkinje burst sizes were maximal), thus erasing the memorised weight patterns. During the synchronisation phase, Purkinje post-complex spike pauses led to a dominant LTP at MF-MVN synapses, reversing the learnt configuration. The interplay between bursts and post-complex spike pauses allowed synaptic adaptation at MF-MVN projections to be highly selective, which resulted in a sparser weight distribution as compared to the case without Purkinje complex spiking (Fig 6A). Therefore, VOR reverse learning required the adjustment of fewer MF-MVN synapses, thus facilitating the eye counteraction of the head velocity movement (S8 Fig), and the weight distribution was reshaped more efficiently with negligible interferences from the previously learnt patterns (Fig 6B and 6C).

### LTP blockades (by dominant LTD) during REMs explain reversal VOR gain discontinuities between training sessions

**Fig 7:**
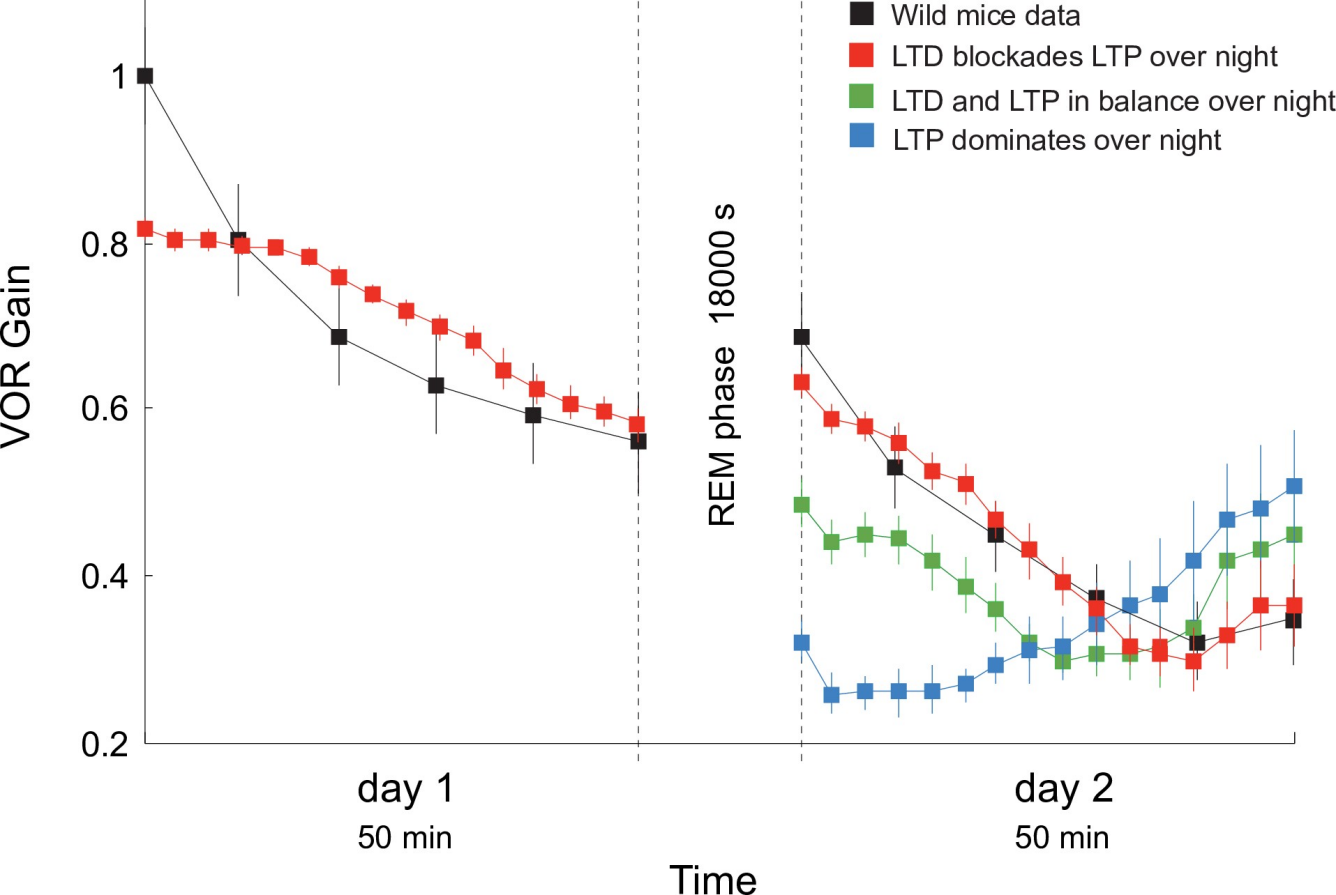
LTP blockades (due to dominant LTD) during REMs explain reversal VOR gain discontinuities between training sessions. We simulated 6 REMs stages (for a total of 18000 s of simulation) between day 1 and 2 of VOR phase-reversal learning. High levels of MF activity (10 Hz) leads to a dominance of LTP at both PF-Purkinje cell and MF-MVN synapses during REMs. Hence, during REMs the cerebellar model keeps ‘forgetting’ the memory traces as during day 1 (blue curve). A smaller MF activity (2.5 Hz) leads to a balance of LTP (driven by vestibular activity) and LTD (driven by the CFs). Thus, the model tends to maintain the synaptic weights learnt during day 1 (green curve). A very low MF activity (1 Hz) makes LTD to block LTP at PF-Purkinje and MF-MVN synapses. Under this third hypothesis, the synaptic weights tend to decay back towards their initial value (red curve) in accordance with experimental data [2] (black curve). See S9 Fig for the modelled probabilistic Poisson process underpinning CF activation.

VOR phase-reversal learning can take place across several days [2] (Fig 5). Dark periods in-between training sessions cause reversal VOR gain discontinuities (Fig 7). This phenomenon has been assumed to result from the decaying of synaptic weights back to their initial values during sleep [2]. However, the mechanisms underlying this decaying process remain unknown. We explored possible cerebellar LTD/LTP balance modulation scenarios occurring during sleep as a consequence of changes in cerebellar activity. During rapid eye movement sleep (REMs), the mean firing activity of Purkinje cells shows increased tonic firing and decreased bursting in both frequency and size [48]. The CF average activity during REMs remains constant at a low frequency regime, showing a tendency in many IO neurons to diminish their overall frequency [49]. The activation of MFs varies during REMs, unrelatedly to any apparent behavioural changes, up to 60 MFs/s on average [49].

We modelled Purkinje cell, CF and MF activities during REMs. CFs were stochastically activated at 1 Hz [48, 49] following a Poisson distribution (S9 Fig). CF activations were also modulated to generate a large event in the Purkinje soma able to elicit bursts of 3 spikes on average [48]. MFs were stochastically activated by mimicking their activity during REMs (with an upper bound firing rate of 8–13 Hz). We tested three hypotheses, based on different levels of cerebellar activity during 6 REMs stages of 3000 s each (i.e., 18000 s of simulation) between days 1 and 2. In the first scenario, we considered high levels of MF activity (average firing rate 10 Hz), which led to a dominance of LTP at both PF-Purkinje cell and MF-MVN synapses during REMs. Consequently, the cerebellar model kept ‘forgetting’ the memory traces as during the reversal VOR learning of day 1 (Fig 7, blue curve). In the second scenario, we considered an average MF activity of 2.5 Hz, which made the LTP driven by vestibular activity counterbalance the LTD driven by the CFs. Under this condition, the cerebellar model consolidated reversal VOR adaptation thus maintaining the synaptic weights at PF-Purkinje and MF-MVN synapses (Fig 7, green curve). Finally, we considered a low level of MF activity (average 1 Hz), which made LTD block the LTP action driven by the vestibular (MF) activity. Under this third scenario, the cerebellar model showed a consistent tendency for weights at PF-Purkinje and MF-MVN synapses to decay back towards their initial values (Fig 7, red curve). Therefore, the model predicts that LTP blockades during REMs stages might underlie the reversal VOR gain discontinuities in-between training sessions, in agreement with experimental data [2] (Fig 7, black curve).

### Purkinje complex spike-pause dynamics under stationary VOR conditions

**Fig 8:**
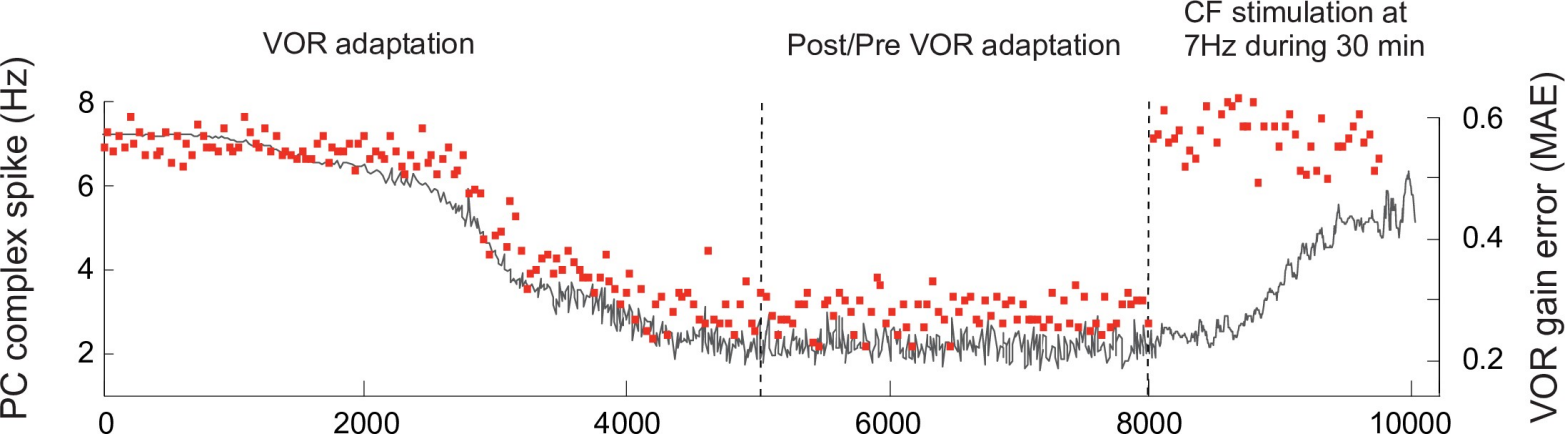
Purkinje complex spike-pause frequency and VOR gain error during adaptation and post/pre adaptation. The frequency of Purkinje complex spike-pauses (red squares) diminishes through VOR adaptation from 8–9 Hz to 2–3 Hz under a sinusoidal vestibular stimulus of ~1 Hz. After VOR adaptation, a direct random stimulation of CFs at 7 Hz during 30 min as in [50] impairs the VOR reflex. The evolution of the VOR gain error (Mean Absolute Error; black curve) during adaptation, post-adaptation, and artificial random stimulation of CFs.

During transient VOR adaptation and phase reversal learning, retina slips were large causing vigorous CF discharges (up to 10 Hz) to encode the sensed errors. Consequently, Purkinje cell complex spike-pauses were elicited at high frequency during adaptation (Fig 8). As the VOR error decreased, the frequency of CF-evoked Purkinje bursts decayed to ~1 Hz upon completion of adaptation (Fig 8). Therefore, during post (and pre) VOR adaptation, model Purkinje tonic Na^+^ spike output dominated and Purkinje cells tended to fire steadily (similar to spontaneous activity) with only rare complex spike-pause firing. Under stationary VOR conditions, (i.e., during pre/post VOR adaptation) model CFs were stochastically activated at ~1 Hz (S9 Fig shows the Poisson-based generative model for the IO firing). Such a CF baseline discharge at ~1 Hz allowed non-supervised LTP to be counterbalanced at PF-Purkinje cell synapses (see Materials & Methods), thus preserving pre/post cerebellar adaptation.

Luebke and Robinson [50] found that directly stimulating CFs at 7 Hz during 30 min after 3 days of VOR adaptation would impair the reflex. Model CFs discharged at frequencies larger than 1 Hz only to signal retina slips (i.e., during VOR adaptation). However, a direct (and error independent) high-frequency stochastic stimulation of CFs would lead to VOR impairment. To illustrate this, we simulated a protocol similar to the one used by [50]. As expected, the number of CF-evoked Purkinje burst-pauses increased as the CF frequency was artificially incremented through a 7 Hz direct stimulation (Fig 8). Therefore, the VOR gain error tended to increase indicating an impairment/blockade of the acquired reflex (Fig 8) and a decrease in VOR gain even with similar CFs discharges observed during VOR adaptation.

## Discussion

Marr and Albus theory [51, 52] elicited a large body of research on the link between behavioural adaptation and the cellular and network properties of the cerebellum. This extensive effort crystallised into a broad range of cerebellar models based on divergent premises. On the one hand, detailed models were grounded on cellular and synaptic properties observed experimentally [53–57]. Most of these biophysical models did not aim at driving behavioural adaptation explicitly through network-level dynamics. On the other hand, numerous large-scale solutions were engineered to be computationally efficient for learning sensorimotor tasks, regardless of the anatomo-functional constraints governing cellular and network cerebellar processes [58–61]. The approach presented here conjugates these two vantage points and focuses on the role of the multiple spiking patterns of Purkinje cells in cerebellar adaptation. It is well known that Purkinje cells can express fast tonic firing as well as a characteristic burst-pause spiking pattern in response to excitatory parallel fibre (PF) and climbing fibre (CF) inputs [44]. Nevertheless, we address here the still uncovered question of how these different spiking patterns regulate the inhibitory action of Purkinje cells onto target medial vestibular nuclei (MVN) and ultimately shape the adaptive behavioural control mediated by the cerebellum.

We model cerebellar-dependent adaptation of the rotational vestibulo-ocular reflex (VOR) (Fig 1A). For natural head rotation frequencies (0.5–5.0 Hz), the VOR gain (i.e., eye velocity divided by head velocity) and the VOR phase shift (i.e., the time lag between eye and velocity profiles) are close to 1 and 180°, respectively [8]. Thus, synchronised counter-phased eye and head movements stabilise visual targets on the fovea, minimising retina slips and improving visual acuity [62]. Cerebellar learning, and particularly Purkinje cell response adaptation, is necessary to mediate online changes in VOR gain control [63, 64]. Thus, numerous VOR models focused on the cerebellar mechanisms at stake during VOR adaptation. *Functional VOR models* capture the input-to-output relationship by abstracting specific cerebellar operations involved during VOR adaptation. Some functional models are derived from the biologically inspired principle of feedback-error learning (FEL) [65, 66], combined with non-parametric statistical learning networks [67, 68]. Other functional VOR models assume that the cerebellum would operate like a bank of recurrent adaptive linear filters supervised by the CF acting as an instructive signal [69, 70]. Another set of functional VOR models use locally weight projection regression (LWPR) algorithms [71] as nonlinear approximators of the granular and molecular cerebellar layers. The output of these LWPR functions is then used as input to Purkinje cells (readout neurons) to control gaze stabilization [72]. *Cellular-level VOR models* capture the features of cerebellar neuronal topology and processing. Amongst these approaches, analogue VOR models (i.e., assuming a neural rate code) can elegantly reproduce behavioural experimental data [2, 26, 73, 74]. Other cellular-level VOR models focus on spatiotemporal spiking representations, by capturing STDP mechanisms as well as Purkinje spike burst-pause dynamics. However, the ability of spiking cerebellar models to cross link cellular, network, and behavioural VOR data remains partly addressed (despite attempts to model and interconnect certain sub-circuits as granular layer [57] or olivary nucleus [75–77]).

The approach presented here belongs to the cellular-level spiking VOR models, and tries to combine neuronal, network, and behavioural description levels. The proposed model mimics the main properties of the cerebellar microcircuit, and it embodies spike-based LTP/LTD plasticity mechanisms at multiple synaptic sites (Fig 1C). At the core of the spiking cerebellar network, a detailed single-compartment model of Purkinje cell reproduces the characteristic tonic, complex spike, and post-complex spike pause patterns [78, 79]. In order to focus on how CF-evoked spike burst-pause dynamics of Purkinje cell responses can regulate the adaptive output of the cerebellum, we also use a simpler Purkinje neuron model that cannot express complex spike firing (i.e., it can only operate in tonic mode). The main finding of this study is that the CF-evoked spike burst-pause dynamics of the Purkinje cell is a key feature for supporting both early and consolidated VOR learning. The model predicts that properly timed and sized Purkinje spike burst-pause sequences are critical to: (1) gating the contingent association between vestibular inputs (about head rotational velocity) and MVN motor outputs (to determine counter-rotational eye movements), mediating an otherwise impaired VOR coarse acquisition; (2) allowing the LTD/LTP balance at MF-MVN synapses to be accurately shaped for optimal VOR consolidation; (3) reshaping previously learnt synaptic efficacy distributions for VOR phase-reversal adaptation. Finally, the model predicts that the reversal VOR gain discontinuities observed after sleeping periods in-between training sessions [2] are due to LTD/LTP balance modulations (and in particular LTP blockades) occurring during REM sleep as a consequence of changes in cerebellar activity.

Our model captures the fact that similar CF discharges occur during both VOR gain increase and decrease adaptation [80, 81]. The direction of retinal slips relative to the vestibular stimulus (assumed to be encoded by CF signals [82]) induces either an increase or a decrease in VOR gain [83]. Interestingly, the relation between CF activity and the induction of plasticity at Purkinje cell synapses is described as gating mechanism that varies under these two VOR adaptation paradigms [81]. Furthermore, optogenetic CF stimulation in VOR gain-decrease paradigms suggest that changes in Purkinje cell complex spike responses do not only depend upon CF activation [81]. Our cerebellar model accounts for these observations by means of the mechanism that balances LTD/LTP plasticity at PF-Purkinje cell synapses. During VOR *gain–increase* adaptation, LTD predominantly blocks LTP at modelled PF-Purkinje cell synapses. This results in a synaptic efficacy decrease as a CF spike reaches the target Purkinje cell (error-related signal). In particular, a CF spike is more likely to depress a PF-Purkinje cell synapse if the PF has been active within 50–150 ms of the CF spike arrival [84–86]. Increasing LTD at PF-Purkinje cell synapses reduces the inhibitory action of Purkinje cells on MVN activity, which in turn, increases the VOR gain. During VOR *gain–decrease* adaptation [29, 80], LTP dominates at PF–Purkinje cell synapses, despite the fact that CF inputs are similar to those occurring during gain-increase phases. A raise in synaptic efficacy at PF-Purkinje cell synapses increases the inhibition of MVN neurons, which in turn, reduces the VOR gain. LTP at modelled PF-Purkinje cell synapses is non-supervised and it strengthens a connection upon each PF spike arrival at the target Purkinje cell. This plasticity mechanism does not need to modulate the input provided by CFs (and then the CF-evoked spike burst-pause dynamics of Purkinje cells) to counter LTD and decrease the VOR gain, in accordance to in-vitro experiments [87–89].

The cerebellar model endowed with CF-evoked Purkinje cell spike burst-pause dynamics performs better, in terms of adaptation accuracy and consolidation rate, than the model with Purkinje cells expressing tonic firing only. CF-evoked spike burst-pause patterns appear particularly useful in a disruptive task such as VOR phase-reversal adaptation. Nevertheless, our results indicate that complex spikes, post-complex spike pauses, and their relative modulation, are not essential for VOR control learning and adaptation. This is in agreement with recent experimental findings challenging the hypothesis that Purkinje cell complex spikes are necessarily required in cerebellar adaptation, and suggesting that their role in motor learning is paradigm dependent [90, 91]. Overall, this work provides insights on how the signals provided by the CFs may instruct, either directly or indirectly, plasticity at different cerebellar synaptic sites [6, 15, 92]. The results point towards a key role of CF-evoked Purkinje cell spike burst-pause dynamics in driving adaptation at downstream neural stages. This testable prediction may help to better understand the cellular-to-network principles underlying cerebellar-dependent sensorimotor adaptation.

### Model assumptions & limitations

This work assumes a gradually modulated CF activity capable of providing an ‘instructive’ signal to Purkinje cells [92]. Evidence exists showing that the presence of the CF signal enables VOR acquisition even in the absence of PF-Purkinje LTD [93], whereas erasing the CF signal impairs VOR adaptation [90]. Nonetheless, the information conveyed by CFs onto Purkinje cells and its potential role in sensorimotor adaptation is under strong debate. The controversy about the nature of CF activity has been further roused by the fact that IO functional properties have so far not been univocally identified [63, 91, 94, 95]. On the one hand, proponents of the Marr-Albus-Ito motor learning theory hypothesise that CFs carry a binary feedback-error signal computed by the IO [96]. Yet, recent studies have questioned the hypothesis of a binary CF signal by demonstrating that the duration of Purkinje cell complex spikes (evoked by CF afferents) can accurately be adjusted based on information that a binary instructive signal could not support [15, 16, 77, 97, 98]. Our model embraces this second hypothesis. On the other hand, despite the CF instructive-role hypothesis is widely accepted in cerebellar learning [6], the overall assumption about IO-mediated feedback-error learning is contrasted by a body of research proposing different roles for the IO rather than coding error [99]. These works focus on the periodic nature of CF activity and they put the CF signalling in relation to the timing aspects of motion [99, 100], and, in particular, to the onset of motion [101]. These counter hypotheses may be classified under five categories: *(i) CFs may act as a temporal information encoder which operates independently of awareness* [102–104]. Subjects were scanned (using event-related functional MRI) whilst observing changes in stimulus timing that were presented near each subject’s detection threshold such that subjects were aware of such changes in only approximately half the trials. The IO and multiple areas within the cerebellar cortex showed a robust response to time changes regardless of whether the subjects were aware of these changes. *(ii) CFs may play a key role in associative somatosensory learning* [105]. In this approach, CFs are thought to provide little or no information about self-produced motion and, therefore, they are not useful for correcting or improving motor performance. Yet, during classical conditioning, the IO may provide the cerebellum with a representation of the unconditioned stimulus for associative learning. *(iii) CF may act as a periodic low-frequency synchroniser [106].* Under this framework, CFs are believed to convey many different types of information, each of which is supposedly assigned to a different narrow time window. Because movement parameters (i.e., end-point error in a cerebellar target-reaching task) are different from trial to trial, it is further hypothesised that a group of CFs innervating a longitudinal synchronous band shall be recruited to convey one particular form of information in each trial at a particular timing. Rhythmicity, randomness, and synchrony could therefore coexist. *(iv) CFs may be responsible for motor timing and reset [107, 108].* Some of the most characteristic morphological features of the olivary neuropil, the glomeruli with their dendrodendritic gap junctions, seem to enable the synchronous activation of clusters of neurons that may act as a temporal clock for motricity. Conversely, subthreshold IO oscillation would allow for a “clock” resetting of a group of neurons. *(v) CFs may be functioning in both motor timing and motor learning [109].* Although synchronous activation of clusters of IO neurons seem to favor the timing hypothesis, it was hypothesised that the olivary micro circuitry (with its unique characteristics, such as the combined excitatory and inhibitory input to the olivary spine) might be able to support both the timing and learning hypotheses, but not the original Marr-Albus-Ito comparator hypothesis.

The cerebellar model presented here assumes a perfect transmission of CF bursts to target Purkinje cells, thus neglecting occasional spike transmission failures observed in vivo [16]. Thus, in the model, there exists a linear relationship between the number of CF stimuli and the length of Purkinje complex spikes. Another limitation is that no distinction between somatic and dendritic spikes is drawn because the Purkinje model consists of a single compartment. Therefore, a key assumption of the model is that CF stimuli elicit both somatic spikes and extra dendritic spikes. Under this assumption, the model predicts a non-linear relation between the length of CF-evoked bursts and the duration of post-complex spike pauses. Indeed, since we adopt a graded representation of the CF instructive signal [15, 19, 28], incremental errors are translated into incremental Purkinje dendritic stimulation intensities. Purkinje calcium-dependent potassium channels activated by Ca2+ influx provoke an after-hyperpolarisation that inhibits the spike generation and modulates the lengthening of the pause [110]. Furthermore, larger numbers of CF stimuli were observed to trigger extra Purkinje dendritic spikes, which influenced Purkinje cell pauses [17, 18]. Also, increasing the number of spikes within the CF burst in the absence of additional dendritic calcium spikes was reported to lead to a decrease in the length of the Purkinje post-complex spike pauses [16]. The model thus assumes a cerebellar operation with a non-linear modulation of the lengths of Purkinje cell post-complex spike pauses due to extra dendritic spikes and large dendritic stimulations during VOR adaptation. Finally, the proposed model considers stationary physiological conditions in the generation of the Purkinje post-complex spike pause (indeed, temperature increase of the cerebellar tissue [111] or delivery of anaesthesia [112] can induce longer, >500 ms, Purkinje simple spike pauses, which ultimately may compromise the spike burst-pause dynamics).

A simplification of our model is that it does not account for the putative role of inhibitory interneurons in the supervised learning mechanism. Understanding the role of this inhibitory network has stimulated numerous experiments and fuelled a debate [113, 114]. It was observed that genetic removal of GABA_A_ receptors of Purkinje cells does not significantly impair mice’s gait and baseline VOR [115], although it does affect the adaptive cerebellar motor control [116]. It was also proposed that this inhibition might regulate the excitatory drive on Purkinje cells by granule cell activity [117]. In the rat cerebellar cortex, GABAergic molecular layer interneurons (which converge on Purkinje cells) are only a small fraction (about 2.3%) of granule excitatory neurons [118]. Overall, these observations point towards the fact that this inhibitory network might not be at the core of the discriminability of input states, whereas it might sub-serve the processes of facilitating cerebellar learning and the correct operation of the cerebellar network [31, 110, 119, 120]. The model suggests that CF-evoked Purkinje cell spike burst-pause dynamics is critical to shape MF-MVN synapses, as to optimise the accuracy and consolidation rate of VOR adaptation. We show that burst and spike pause sequences facilitate sparser MF-MVN connections, which increases coding specificity during the adaptation process. The results predict that the spike burst-pause dynamics should be central to retune MF-MVN synapses during VOR phase-reversal adaptation. First, it is shown that blocking complex spike responses (and post-complex spike pauses) in Purkinje cells impairs reverse VOR adaptation. More strikingly, the results indicate that Purkinje cell bursting and spike pauses ensure the reversibility of the adaptation process at MF-MVN synapses. Bursts selectively facilitate LTD at MF-MVN connections, which rapidly erases previously learnt memory traces at these synapses. Subsequently, post-complex spike pauses induce strong LTP at MF-MVN synapses, which allows the cerebellar output to become rapidly reverse-correlated to the sensed error. In addition, the memory consolidation of VOR adaptation during sleeping [2, 73, 121] is also supported by the CF-evoked Purkinje cell spike burst-pause dynamics. CF stochastically activations at a low frequency (0.9 Hz) during REMs stages maintain a base Purkinje bursting that ultimately facilitates LTP blockades at PF-Purkinje cell and MF-MVN synapses, and it preserves the on-going learning process.

## Materials and methods

### VOR analysis and assessment

We simulated horizontal VOR (h-VOR) experiments with mice undertaking sinusoidal (~1 Hz) whole body rotations in the dark [40]. The periodic functions representing eye and head velocities (Fig 1A) were analysed through a discrete time Fourier transform. The **VOR gain** was calculated as the ratio between the first harmonic amplitudes of the forward Fourier eye–and head–velocity transforms:

$$VOR\;GAIN\;G=\frac{A_{1}^{eye-velocity}}{A_{1}^{head-velocity}}$$ (1)

In order to assess the **VOR shift phase**, the cross-correlation of the eye and head velocity time series was computed:

$$xcorr=\left(x*y\right)\left[\gamma \right]\;\overset{def}{=}\sum_{n=-\infty }^{+\infty }x^{*}\left(n\right)y\left(n+\gamma \right)$$ (2)

where x^*^ is the complex conjugate of *x*, and γ the lag (i.e., shift phase). The ideal eye and head velocity lag is ±0.5 after normalisation, with cross-correlation values ranged within [–1, 1], which is equivalent to a phase shift interval of [–360° 360°].

### Simulation of VOR protocols

We simulated a rotational chair test, in which a subject (mouse, monkey, or human) is seated in a rotatory table. In this protocol, the velocity of rotation is controlled and the subject’s head is restrained, assuming that the movement of the table is equal to the subject’s head movement. During **normal VOR adaptation**, a visual target is provided in anti-phase with vestibular stimulation. The eyes must follow the visual target, thus minimising retinal slips. The vestibular stimulation and the eye output functions in our simulation were taken as:

$$\begin{array}{l} Vestibular\;stimulation=sin\left(2\cdot \pi \cdot t\right)\\ Eye\;output\;function=A_{E}\cdot sin\left(2\cdot \pi \cdot t+\pi \cdot \phi_{E}\right) \end{array}$$ (3)

where the ideal VOR experiment values correspond to $A_{E}=1,\phi_{E}=0$ (visual field fixed). During **VOR phase-reversal learning,** the visual stimulus is given in-phase with the visual field but it turns twice the distance of the turntable, i.e. *A*_*E*_ *= -1*.

### Cerebellar spiking neural network model

The cerebellar circuit was modelled as a feed–forward loop capable of compensating head movements by producing contralateral eye movements (Fig 1B). The connectivity and the topology of the simulated cerebellar network involved five neural populations: mossy fibres (MFs), granule cells (GCs), medial vestibular nuclei (MVN), Purkinje cells, and inferior olive (IO) cells [33, 122–125]. During simulated 1 Hz head rotations, sensorimotor activity was translated into MF activity patterns that encoded head velocity. MFs transmitted this information to both MVN and GCs. The latter generated a sparse representation of head velocity signals, which was sent to Purkinje cells through the PFs. Purkinje cells were also driven by the CFs, which conveyed the instructive signal encoding sensory error information (i.e., retina slips due to the difference between actual and target eye movements, [82]). Finally, Purkinje cells’ output inhibited MVN neurons, which closed the loop by shaping cerebellar-dependent VOR control. The CF-Purkinje cell-MVN subcircuit was divided in two symmetric micro-complexes for left and right h-VOR, respectively. The input-output function of the cerebellar network model was made adaptive through spike-timing dependent plasticity (STDP) at stake at multiple sites (Fig 1C). These STDP mechanisms led to both long-term potentiation (LTP) and long-term depression (LTD) of the ~50000 synapses of the cerebellar model see [126]. This spiking neural network model was implemented in EDLUT [86, 127, 128] an efficient open source simulator mainly oriented to real time simulations.

### Purkinje cell model

We used a detailed Purkinje cell model based on the experimental work by Middelton et al. [78], and on the modelling work by Miyasho et al. [79]. The model consisted of a single compartment with five ionic currents:

$$\frac{dV}{dt}=-g_{K}\cdot n^{4}\cdot \left(V+95\right)-g_{Na}\cdot m_{0}{\left[V\right]}^{3}\cdot h\cdot \left(V-50\right)--g_{Ca}\cdot c^{2}\cdot \left(V-125\right)-g_{L}\cdot \left(V+70\right)-g_{M}\cdot M\cdot \left(V+95\right)$$ (4)

with $g_{K}$ denoting a delayed rectifier potassium current, $g_{Na}$ a transient inactivating sodium current, $g_{Ca}$ a high-threshold non-inactivating calcium current, $g_{L}$ a leak current, and $g_{M}$ a muscarinic receptor suppressed potassium current (see Table 1).

**Table 1:** Ionic conductance densities.

*Conductance type*	*Soma (mho/cm2)*
*g*_*K*_*–delayed rectifier potassium current*	*0*.*01*
*g*_*Na*_*–transient inactivating sodium current*	*0*.*125*
*g*_*Ca*_*–high threshold*	*0*.*001*
*g*_*M*_*–muscarinic receptor*	*0*.*75*
*g*_*L*_*–leak current (anomalous rectifier)*	*0*.*02*

The dynamics of each gating variable evolved as follows:

$$\overset{\cdot }{x}=\frac{x_{0}[V]-x}{\tau_{x}[V]}$$ (5)

where x indicates the variables n, h, c, and M. The implemented equilibrium function is determined by the term $x_{0}\left[V\right]$ and time constant $\tau_{x}\left[V\right]$ (Table 2).

**Table 2:** Ionic conductance kinetic parameters.

***Conductance type***	***Steady–state*** ***Activation/Inactivation***		***Time constant (ms)***
*g*_*K*_*–delayed rectifier potassium current*	*$x_{0}\left[V\right]=\frac{1}{1+e^{\frac{-V-29.5}{10}}}$*		*$\tau_{x}\left[V\right]=\left\{ \begin{array}{l} 0.25+4.35\cdot e^{\frac{V+10}{10}}\;if\;V\leq -10\\ 0.25+4.35\cdot e^{\frac{-V-10}{10}}\;if\;V>-10 \end{array}\right.$*
*g*_*Na*_*–transient inactivating sodium current*	*$x_{0}\left[V\right]=\frac{1}{1+e^{\frac{V+59.4}{10.7}}}$*		*$\tau_{x}\left[V\right]=0.15+\frac{1.15}{1+e^{\frac{V+33.5}{15}}}$*
$m_{0}\left[V\right]$	*$m_{0}\left[V\right]=\frac{1}{1+e^{\frac{-V-48}{10}}}\cdot m$*		
	***Forward Rate Function(α)***		***Backward Rate Function(β)***
*g*_*Ca*_*–high threshold*	*$\alpha =\frac{1.6}{1+e^{-0.0072\cdot \left(V-5\right)}}$*		*$\beta =\frac{0.02\cdot \left(V+8.9\right)}{e^{\frac{V+8.9}{5}}-1}$*
*g*_*M*_*–muscarinic receptor suppressed potassium current*	*$\alpha =\frac{0.3}{1+e^{\frac{-V-2}{5}}}$*		*$\beta =0.001\cdot e^{\frac{-V-70}{18}}$*
	***Steady–state*** ***Activation/Inactivation***		***Time constant(ms)***
	*$x_{0}\left[V\right]=\frac{\alpha }{\alpha +\beta }$*		*$\tau_{x}\left[V\right]=\frac{1}{\alpha +\beta }$*

The sodium activation variable was replaced and approximated by its equilibrium function $m_{0}\left[V\right]$. M-current presents a temporal evolution significantly slower than the rest of the five variables thus provoking a slow-fast system able to reproduce the characteristic Purkinje cell spiking modes (Fig 2).

The final voltage dynamics for the Purkinje [78, 79]cell model was given by:

$$\frac{dV}{dt}=\frac{-g_{K}\cdot n^{\text{4}}\cdot (V+\text{95})-g_{Na}\cdot m_{0}{\left[V\right]}^{3}\cdot h\cdot (V-50)-g_{Ca}\cdot c^{2}\cdot (V-125)-g_{L}\cdot (V+70)-g_{M}\cdot M\cdot (V+95)+\frac{Injected\;Current}{Membrane\;Area}}{Membrane\;Capacitance}$$ (6)

where the parameters *Membrane Area* and *Membrane Capacitance* are provided in Table 3, and *Injected Current* is the sum of all contributions received through individual synapses (see Eqs 7–9 below).

**Table 3:** Geometrical parameters.

*Geometrical parameters*	
Cylinder length of the soma	15*μm*
Radius of the soma	8*μm*
Membrane capacitance	1*μF/cm*^*2*^
Axial resistivity	100 _*Ω-CM*_(axon) 250_*Ω-CM*_(dendrites)
Number of segments	1

First, we validated the detailed Purkinje cell model (Eqs 4–6) in the Neuron simulator. Subsequently, we reduced the Purkinje cell model to make it compatible with an event-driven lookup table simulator (i.e, the EDLUT simulator) for fast spiking neural network simulations [86, 127]. In the reduced Purkinje cell model, *I*_*K*_ and *I*_*Na*_ currents were implemented through a simple threshold process that triggers the generation of a triangular voltage function each time the neuron fires [129]. This triangular voltage depolarisation drives the state of ion channels similarly to the original voltage depolarisation during the spike generation. We inserted the differential equation defined in Eq 6 within EDLUT. We used an in-house fixed-step numerical integration method compatible with GPUs (bi-fixed integrative method). Our numerical integration method provided similar accuracy than the variable step-size numerical integration methods provided by NEURON with considerably less computational cost [128]. To make NEURON simulation comparable with EDLUT as in S1 Fig, the stimulation of the Purkinje cell was carried out by spike trains through an AMPA synapse (single decaying exponential with τ = 1 ms, E_exc_ = 0 mV). We emulated the effect of the PF over the Purkinje cell through a spike train of 55 Hz and a synaptic weight of g_exc_ = 8 μS. We used CF synaptic stimulations through an AMPA synapse with weight of g_exc_ = 80 μS, (see asterisks in Fig 2C). The spike timing traces of the Purkinje spike burst-pause dynamics under equal Purkinje stimulation were consistent in NEURON and EDLUT. Both the EDLUT and NEURON source codes are available at the following URLs:

www.ugr.es/~nluque/restringido/Burst-pause_Purkinje_dynamics_regulate_motor_adaptation_NEURON_MODEL_COMPLETE.rar

www.ugr.es/~nluque/restringido/CODE_Burst-pause_Purkinje_dynamics_regulate_motor_adaptation_EDLUT.rar

User: REVIEWER, password: REVIEWER (for both).

### Other cerebellar neuron models

The other cerebellar neurons (granule cells, MVN cells, …) were simulated as leaky integrate–and–fire (LIF) neurons, with excitatory (AMPA) and inhibitory (GABA) chemical synapses:

$$C_{m}\cdot \frac{dV_{m-c}}{dt}=g_{AMPA}\left(t\right)\cdot \left(E_{AMPA}-V_{m-c}\right)+g_{GABA}\left(t\right)\cdot \left(E_{GABA}-V_{m-c}\right)+G_{rest}\cdot \left(E_{rest}-V_{m-c}\right)$$ (7)

where *C*_*m*_ denotes the membrane capacitance, *E*_*AMPA*_ and *E*_*GABA*_ are the reversal potential of each synaptic conductance, *E*_*rest*_ is the resting potential, and *G*_*rest*_ indicates the conductance responsible for the passive decay term towards the resting potential. Conductances *g*_*AMPA*_ and *g*_*GABA*_ integrate all the contributions received by each receptor type (AMPA and GABA) through individual synapses and they are defined as decaying exponential functions [86, 130]:

$$g_{AMPA}\left(t\right)=\left\{ \begin{array}{l} 0\;\;\;\;\;\;\;\;,\;t\leq t_{0}\\ g_{AMPA}\left(t_{0}\right)\cdot e^{-\frac{\left(t-t_{0}\right)}{\tau_{AMPA}}},\;t>t_{0} \end{array}\right.\;$$ (8)

$$g_{GABA}\left(t\right)=\left\{ \begin{array}{l} 0\;\;\;\;\;\;\;\;,\;t\leq t_{0}\\ g_{GABA}\left(t_{0}\right)\cdot e^{-\frac{\left(t-t_{0}\right)}{\tau_{GABA}}},\;t>t_{0} \end{array}\right.\;$$ (9)

with *t* representing the simulation time, *t*_*0*_ being the time arrival of an input spike, and *τ*_*AMPA*_ and *τ*_*GABA*_ denoting the decaying time constant for AMPA and GABA receptors, respectively.

Note that we also used the LIF neuronal model (Eqs 7–9) to simulate Purkinje cells that could only express tonic spike firing (Fig 3B). These Purkinje cells with compromised CF-evoked spike burst-pause dynamics provided a coarse phenomenological model of Kv3.3-deficient Purkinje neurons (as in Kcnc3 mutants, in which the absence of voltage-gated potassium channel Kv3.3 drastically reduces spikelet generation within complex spikes of cerebellar Purkinje cells) [131]. Note, however, that completely suppressing CF-evoked spike burst-pause dynamics would require more severe actions. A plausible way for obtaining no-bursting-after-CF stimulus may consist in modulating GABA_B_ Purkinje cell receptors via Baclofen, the specific GABA_B_ agonist (as shown in [132]). Table 4 summarises the parameters used for each cell and synaptic receptor type.

**Table 4:** Parameters of the LIF cell types.

*Parameter*	*Granule Cell*	*Purkinje LIF Cell*	*MVN Cell*
*Refractory period*	*1ms*	*2ms*	*1ms*
*Membrane capacitance*	*2pF*	*40pF*	*2pF*
^*^Total excitatory *peak conductance*	*1nS·100*	*1*.*3nS·* ·175000·10%^*^	*1nS·7*
*Total inhibitory* *peak conductance*	*1nS·200*	*3nS·150*	*30nS·1*
*Threshold*	*–40mV*	*–52mV*	*–40mV*
*Resting potential*	*–70mV*	*–70mV*	*–70mV*
*Resting conductance*	*0*.*2nS*	*1*.*6nS*	*0*.*2nS*
*Resting* *time constant (τ*_*rest*_*)*	*10ms*	*25ms*	*10ms*
*Excitatory–synapse* *time constant (τ*_*AMPA*_*)*	*0*.*5ms*	*0*.*5ms*	*0*.*5ms*
*Inhibitory–synapse* *time constant (τ*_*GABA*_*)*	*10ms*	*1*.*6ms*	*10ms*

Parameters obtained from the following papers:

Granule cell (GC)[133–137]. Only the rapidly decaying component of AMPA is modelled (τ_AMPA=0.5ms_) the presence of slowly decaying components in some GC caused by spill overs of glutamate was not taken into consideration [133](τ_AMPA=3ms_)[138] Purkinje cell (PC) [137, 139–141]. MVN data were extracted from unpublished material from Prof. D’Angelo’s lab.

* Where 10% means the ratio of active connections PF–PC (out of the total 175000 PFs)

### Cerebellar neural population models

#### Mossy fibres (MFs)

N = 100 MFs were modelled as LIF neurons (Eqs 7–9). Consistently with the functional principles of VOR models of cerebellar control [2], the ensemble MF activity was generated following a sinusoidal shape (1 Hz with a step size of 0.002 ms) to encode head movements [2, 142, 143]. The overall MF activity was based on non-overlapping and equally sized neural subpopulations that allowed a constant firing rate of the ensemble MFs to be maintained over time. Importantly, two different times always corresponded to two different subgroups of active MFs ensuring the overall constant activity. (Network connectivity parameters summarised in Table 5).

**Table 5:** Summary of neurons and synapses.

*Neurons*			*Synaptic weights (nS)*		
*Presynaptic cell number*	*Postsynaptic cell*	*Number of synapses*	*Type*	*Initial weight (Detailed/non Detailed PC)*	*Weight range*
Mossy Fibres (100)	Granular Cells	8000	AMPA	0.35/0.35*	
	Medial Vestibular Nuclei	200	AMPA	0.0/0.0	[0, 10] /[0, 10]
Climbing Fibres (2)	Purkinje Cells	20	AMPA	40/2.5	
Granular Cells (1000)	Purkinje Cells	40000	AMPA	3.4/3.75	[0, 3.75] / [0, 5.5]
Purkinje cell (20)	Medial Vestibular Nuclei	20	GABA	0.15/0.15	[0 10] / [0, 10]
Medial Vestibular Nuclei (2)					

* Parameter used for generating the Granular layer activity. Since this activity remained invariant during VOR adaptation, it was stored offline in a file and then loaded in computation time.

#### Granular cells (GCs)

The granular layer included N = 2000 GCs and it was implemented as a state generator [144–147], i.e. its inner dynamics produced time–evolving states even in the presence of a constant MF input **[59].** The granular layer generated non-overlapped spatiotemporal patterns that were repeatedly activated in the same sequence during each learning trial (1 Hz rotation for 1 s)). 500 different states encoded each second of the 1 Hz learning trial, each state consisting of four non-recursively activated GCs.

#### Climbing fibres (CFs)

N = 2 CFs carried the instructive signal (from the IO) to the population of Purkinje cells. The two CFs handled clockwise and counter–clockwise sensed errors. CF responses followed a probabilistic Poisson process. Given the normalised error signal ε(t) and a random number  η(t) between 0 and 1, a CF fired a burst if ε(t)>η(t), otherwise it remained silent [84, 120, 148]. Thus, a single CF burst encoded time information regarding the instantaneous error. Furthermore, the probabilistic spike sampling of the error ensured a proper representation of the whole error region over trials, whilst maintaining the CF activity below 10 Hz per fibre (similar to electrophysiological data; [149]). The evolution of the sensed error could be sampled accurately even at such a low frequency [148, 150]. A graded representation of the instructive signal [15, 19, 28] enabled the correlation between the intensity of the sampled instantaneous error and the number of the spikes within the olivary burst:

$$\begin{array}{l} S_{spikes}:[0,1]\underset{\varepsilon \to y=S_{spikes}(\varepsilon )}{\subseteq \mathbb{R}\to \mathbb{R}}\\ S_{spikes}(\varepsilon )=\{\begin{array}{ccc} 2 & if & 0.25\leq \varepsilon \leq 0.50\\ 3 & if & 0.50\leq \varepsilon \leq 0.75\\ 4 & if & 0.75\leq \varepsilon \leq 0.85\\ 5 & if & 0.85\leq \varepsilon \leq 0.95\\ 6 & if & 0.95\leq \varepsilon \leq 1.0 \end{array} \end{array}$$ (10)

The model assumed a perfect linearly correlated transmission of olivary bursts from CFs to the target Purkinje cells. Hence, the number of spikes in the Purkinje complex spikes linearly depended on the number of stimuli in the CF burst [16]. CFs were simulated to transmit from 2 to 6 CF stimuli, delivered at inter-stimulus intervals of 2 ms [16], signalling the sensed error to be compensated. For the sake of computational efficiency, only 2 CFs were simulated (instead of 20). In the cerebellum, each Purkinje cell is innervated by a single CF [151] coming from the associated IO in the olivary system. However, no olivary system was considered here and, consequently, CFs sensing clockwise and counter–clockwise errors were equally activated (it would suffice 1 CF sensing clockwise and 1 CF sensing anti-clockwise errors).

#### Purkinje cells

N = 20 Purkinje cells were divided in two subpopulations of 10 neurons each. Each subpopulation received the inputs from one CF encoding the difference between (either rightward or leftward) eye and head movements. Each Purkinje cell also received 2000 PF inputs. Since real Purkinje cells are innervated by about 150000 PFs [152], the weights of the PF–Purkinje cells synapses of the model were scaled so as to obtain a biologically plausible amount of excitatory drive. Each of the two subgroups of 10 Purkinje cells targeted (through inhibitory projections) one MVN cell, responsible for either clockwise or counter-clockwise compensatory motor actions (ultimately driving the activity of agonist/antagonist ocular muscles).

#### Medial vestibular nuclei (MVN)

The activity of N = 2 MVN cells produced the output of the cerebellar model. The two MVN neurons handled clockwise and counter–clockwise motor correction, respectively. Each MVN neuron received excitatory projections from all MFs (which determined the baseline MVN activity), and inhibitory afferents from the corresponding group of 10 Purkinje cells (i.e., the subcircuit IO–Purkinje cell–MVN was organised in a single microcomplex).

#### Translation of MVN spike trains into analogue eye motor commands

MVN spike trains were translated into analogue output signals through a Finite Impulse Response filter (FIR) [153]. Let $x\left(t\right)=\sum_{j=t}^{M}\delta \left(t-t_{j}\right)$ denote a MVN spike train, with *t*_*j*_ being the firing times of the corresponding neuron. If *h(t)* indicates the FIR kernel, then the translated MVN output is:

$$Output\left(t\right)=\left(h*x\right)\left(t\right)=\sum_{j=t}^{M}h\left(t-t_{j}\right)$$ (11)

Note that a delay is introduced in the generated analogue signal. This delay is related to the number of filter coefficients and to the shape of the filter kernel *h(t)*. In order to mitigate this effect, we used an exponentially decaying kernel:

$$Kernel=h\left(t\right)=e^{-\frac{M}{\tau_{M}}}$$ (12)

where *M* is the number of filter taps (one per integration step) and *τ*_*M*_ is a decaying factor. At each time step, the output signal value only depends on its previous value and on the input spikes in the same time step. Therefore, this filter is implemented by recursively updating the last value of the output signal. Importantly, this kernel is similar to postsynaptic current functions [154, 155], thus facilitating a biological interpretation. Furthermore, this FIR filter is equivalent to an integrative neuron [156]. The final eye movement is taken proportional to the inverse value of *Output(t)*.

### Synaptic plasticity rules

#### PF–Purkinje cell synaptic plasticity

The LTD/LTP balance at PF–Purkinje cell synapses was based on the following rule (S2 Fig shows sensitivity analyses accounting for LTD/LTP balance):

$$\begin{array}{l} LTD.\Delta w_{PF_{j}-PC_{i}}\left(t\right)=\int_{-\infty }^{IO_{spike}}k\left(\frac{t-t_{IO_{spike}}}{\tau_{LTD}}\right)\cdot \delta_{GC_{spike}}\left(t\right)\cdot dt\;if\;PF_{j}\;is\;active\;at\;\;t\\ LTP.\Delta w_{PF_{j}-PC_{i}}\left(t\right)=\alpha \cdot \delta_{GC_{spike}}\left(t\right)\;const.\;otherwise \end{array}$$ (13)

where *ΔW*_*PFj–PCi*_*(t)* denotes the weight change between the *j*^*th*^ PF and the target *i*^*th*^ Purkinje cell; t*LTD* is the time constant that compensates for the sensorimotor delay (100ms); ð*GCspike(t)* is the Dirac delta function corresponding to an afferent spike from a PF (i.e., emitted by a GC); and the kernel function *k(x)* is defined as [126]:

$$k\left(x\right)=e^{-x}\cdot \sin{\left(x\right)}^{20}$$ (14)

The convolution in Eq 14 was computed on presynaptic PF spikes arriving 100 ms before a CF spike arrival, accounting for the sensorimotor pathway delay [7, 96, 120, 148, 157]. Note that the kernel *k(x)* allows the computation to be run on an event–driven simulation scheme as EDLUT [86, 120, 148, 157], which avoids integrating the whole kernel upon each new spike arrival. Finally, as shown in Eq 13, the amount of LTP at PF–Purkinje cell synapses was fixed, with an increase in synaptic efficacy equal to α each time a spike arrived through a PF to the target Purkinje cell.

#### MF–MVN synaptic plasticity

The LTD/LTP dynamics at MF-MVN synapses was taken as (S2 Fig shows sensitivity analyses accounting for LTD/LTP balance):

$$\begin{array}{l} LTD.\Delta w_{MF_{j}-MVN_{i}}\left(t\right)=\int_{-\infty }^{+\infty }k\left(\frac{t-t_{PC_{spike}}}{\sigma_{MF-MVN}}\right)\cdot \delta_{MF_{spike}}\left(t\right)\cdot dt\;if\;PC_{j}\;is\;active\;at\;\;t\\ LTP.\Delta w_{MF_{j}-MVN_{i}}\left(t\right)=\alpha \cdot \delta_{MF_{spike}}\left(t\right)\;const.\;otherwise \end{array}$$ (15)

with *ΔW*_*MFj–MVNi(t)*_ denoting the weight change between the *j*^*th*^ MF and the target *i*^*th*^ MVN. $\sigma_{MF-DCN}$ standing for the temporal width of the kernel; $\delta_{MF}$ representing the Dirac delta function that defines a MF spike; and the integrative kernel function *k(x)* defined as [126]:

$$k\left(x\right)=e^{-\left|x\right|}\cdot \cos{\left(x\right)}^{2}$$ (16)

Note that there is no need to compensate the sensorimotor pathway delay at this site because it is already done at PF-Purkinje cell synapses (*τ*_*LTD*_ in Eq 13).

The STDP rule defined by Eq 15 produces a synaptic efficacy decrease (LTD) when a spike from the Purkinje cell reaches the targeted MVN neuron. The amount of synaptic decrement (LTD) depends on the activity arrived through the MFs. This activity is convolved with the integrative kernel defined in Eq (16). This LTD mechanism considers those MF spikes that arrive after/before the Purkinje cell spike arrival within the time window defined by the kernel. The amount of LTP at MF-MVN synapses is fixed (Ito, 1982;[126, 158], with an increase in synaptic efficacy each time a spike arrives through a MF to the targeted MVN.

#### Purkinje cell–MVN synaptic plasticity

The STDP mechanism implemented at Purkinje cell-MVN synapses [126] consists of a traditional asymmetric Hebbian kernel

$$\begin{array}{l} \Delta w_{PC_{j}-MVN_{i}}\left(t\right)=\left\{ \begin{array}{l} LTP\cdot e^{-\frac{t_{MVN\_post}-t_{MVN\_pre}}{\sigma_{PC-MVN}^{+}}}\;if\;t_{MVN\_post}\geq t_{MVN\_pre}\\ LTD\cdot e^{-\frac{t_{MVN\_pre}-t_{MVN\_post}}{\sigma_{PC-MVN}^{-}}}\;otherwise \end{array}\right. \end{array}$$ (17)

where *ΔW*_*PCj–MVNi(t)*_ is the weight change between the *j*^*th*^ PC and the target *i*^*th*^ MVN, $\sigma_{PC-MVN}^{+}$ and $\sigma_{PC-MVN}^{-}$ are the time constants of the potentiation and depression components set to 5ms and 15ms respectively; and LTD_max_/LTP_max_ (0.005/0.005) are the maximum weight depression/potentiation change per simulation step. The t_mvn_post_ and t_mvn_pre_ indicate the postsynaptic and presynaptic MVN spike time. This STDP rule is consistent with the fact that plasticity at Purkinje cell-MVN synapses depends on the intensity of MVN and Purkinje cell activities [23–26] and it provides a homeostatic mechanism in balancing the excitatory and inhibitory cell inputs to MVN [124, 159].

## Supporting information

S1 Fig Purkinje cell pause duration. **(A)** In the model, CF signals modulate both the burst size (i.e., the number of spikes within the burst [17, 18]) and the duration of post-complex spike pause. **(B)** Across multiple simulations, we progressively increased the size of CF burst stimulation: from 4 ms (i.e., 2 spikes) to 12 ms (i.e., 6 spikes), by steps of 2 ms. For each of the 5 stimulation conditions, we varied the depolarisation current injected through PFs to elicit Purkinje responses within their operative frequency range (i.e., 50–250 Hz). We then used a Kruskal-Wallis test to assess the relationship between Purkinje spike pause lengths and CF burst duration. We found a statistically significant difference (Chi square = 145.61, p<10^−20^, df = 4) amongst the five conditions (i.e. CF burst sizes: 4, 6, 8, 10, and 12 ms). A Bonferroni post hoc test revealed that only the conditions 4 ms and 6 ms produced significantly shorter pauses, whereas the non-linear relation plateaued from 6–8 to 12 ms. **(C)** In the Purkinje cell model, the CF stimulation–CS pause length relationship is mediated by the muscarinic receptor channel. We simulated a random modulation of the time constant of the muscarinic receptor ion channel to generate stochastic Purkinje post-complex spike pauses (i.e. independently from CF stimulation). To do so, we multiplied the time constant of the muscarinic channel by a random factor at each time step (0.002 ms). Hence, whilst the activation/inactivation of the muscarinic channel remained unaltered, therefore maintaining Purkinje spike bursting, the duration of pauses was randomly modulated. The modified Purkinje cell model was used to run the same series of simulations as in B by gradually increasing the CF burst size (i.e., 4, 6, 8, 10, 12 ms). The Kruskal-Wallis test confirmed that the inserted stochastic mechanism removed any correlation between the length of Purkinje spike pauses and the CF burst sizes (Chi square = 4.06, p = 0.398, df = 4; S1C Fig). The model with random length post-complex spike pauses was then compared against the original model (B) in terms of performance in VOR adaptation (S7 Fig).
(PDF)

S2 Fig Critical LTD/LTP balance at PF-Purkinje cell and MF-MVN synapses. **Parameter sensitivity analysis.** Cerebellar adaptation modulates PF-Purkinje cell synaptic weights as well as MF-MVN synapses [6, 126]. For synaptic adaptation, the model uses supervised STDP, which exploits the interaction amongst unsupervised and supervised cell inputs to regulate and stabilise postsynaptic activity. Balancing supervised STDP, and the resulting synaptic modification dynamics, is critical, given the high sensitivity of the process that determines the LTD/LTP ratio [160, 161]. A sensitivity analysis of the parameters governing LTD and LTP, shows that LTP exceeding LTD values for a narrow range at MF-MVN synapses preserves VOR learning stability. This holds independently for both VOR gain and phase **(A)** as well as for the combination of the two **(B)**. By contrast, PF-Purkinje cell synapses admit broader limits for the LTD/LTP ratio (A, B). *Detailed description*: we systematically simulated LTP/LTD ratio values at PF-Purkinje cell and MF-MVN synapses within a plausible range that may satisfy the expected h-VOR outcome. As simulations ran, the solutions were iteratively checked until finding the set of LTD/LTP ratio values that exhibited the better performance in terms of h-VOR gain and phase. LTD/LTP balance at each site was modified by systematically multiplying LTD by 1.5^N^ where –11 ≤ N ≤ 12 for PF-Purkinje cell and MF-MVN synapses. For each parameter setting, the cerebellar model underwent 10 000 s of VOR learning (1Hz head rotation movement to be compensated by contralateral eye movements. **(A)** Final VOR gain and phase plotted over the LTD/LTP range of values that were tested. **(B)** Combined VOR gain and phase (normalised) as a function of the LTD/LTP ratio. At PF-Purkinje cell synapses the LTD/LTP was well balanced for N values ranging between [–1, 7]. At MF-MVN the LTD/LTP balance was more critical since N is within a narrower band range [–1, 0]. The reddish area within the last plot indicates the optimal parameters range. LTP must exceed LTD at MF-MVN synapses for optimal VOR performance. This result is consistent with the unsupervised nature of the LTP for the kernel defined for MF-MVN STDP. Unsupervised LTP with larger values than LTD takes the MF-MVN synaptic weights to the upper bound of their synaptic efficacy, thus provoking more MVN activations. In the absence of LTD counteraction, the cerebellar output is, therefore, upper saturated. LTD driven by Purkinje cell activity blocks LTP at MF-MVN synapses, thus shaping the cerebellar compensatory output.
(PDF)

S3 Fig LTD/LTP balance at MF-MVN synapses over time. Whilst LTD/LTP balance was fixed at PF-PC synapses, we modified the LTD/LTP balance at MF-MVN synapses by systematically varying the ratio by 1.5^N^ where –11 ≤ N ≤ 12 during a 10000 s simulation. **(A)** Final VOR gain and phase plotted as a function of the tested LTD/LTP range across time. **(B)** Combined VOR gain and phase (normalised) over time. A proper balance between LTD and LTP (ratio of approximately 0.4) makes the cerebellum perform optimally after 750 sec.
(PDF)

S4 Fig Parameter sensitivity analysis for the LTD/LTP balance at PF-Purkinje cell and MF-MVN synapses in the absence of Purkinje spike burst-pause dynamics. Similar to S2 Fig, the parameters regulating the LTD/LTP ratio were exhaustively tested whilst the cerebellar model without Purkinje complex spiking underwent h-VOR learning during a 10000 s simulation. **(A)** Final VOR gain and phase plotted over the LTD/LTP range of tested values. **(B)** Combined VOR gain and phase (normalised) as a function of the LTD/LTP ratio. LTD/LTP at both PF-Purkinje cell synapses is well balanced for N values ranged between [–1, 7]. Thus, the absence of bursting and pause dynamics leads to a wider range values for the LTD/LTP balance.
(PDF)

S5 Fig VOR gain of nine subjects [162] vs. VOR gain obtained from the cerebellar model tested at multiple frequencies during passive head rotations. Average VOR gain/phase calculated by taken gain values each 400 s over the last 4000 s of the VOR adaptation process (10000 s) over a range of frequencies within the natural head rotation range [0.05-5Hz]. Consistently with the known frequency spectrum of the vestibular system [163], VOR gain remained relatively stable across the tested frequencies.
(PDF)

S6 Fig VOR phase-reversal learning. **Time course of the VOR phase**. **(A)** VOR phase adaptation with (red curve) and without (green curve) Purkinje spike burst-pause dynamics. **(B)** Focus is on the phase-reversal period and comparison with experimental data [2].
(PDF)

S7 Fig The presence of Purkinje post-complex spike pauses is relevant to VOR adaptation. VOR gain adaptation mediated by the model with Purkinje spike burst-pause dynamics (orange and red curves, with stochastic vs. burst-dependent pause lengths, respectively; S1 Fig) and by the model without spike burst-pause dynamics (green curve). The simulated protocol is the same of Fig 5: VOR adaptation (first 10 000 s), phase-reversal learning (subsequent 12 000 s), and VOR restoration (remaining 12000 s). The presence of Purkinje spike burst-pause dynamics, regardless the relation between CF burst sizes and pause lengths (S1 Fig), improves the performance of cerebellar-dependent VOR adaptation.
(PDF)

S8 Fig Eye velocity evolution during VOR phase-reversal learning. **(A**) Only the eye velocity movement corresponding to the sparser and more selective distribution of MF-MVN synaptic weights is able to counteract the head velocity movement in counter phase (**B**), as phase-reversal learning is achieved (**C**).
(PDF)

S9 FigClimbing fibre activation.In the model, CF responses follow a probabilistic Poisson process. Given the normalised error signal ε(t) obtained from the retina slip and a random number  η(t) between 0 and 1, the model CF fires a spike if ε(t)>η(t); otherwise, it remains silent[84] A single spike is then able to report timed information regarding the instantaneous error. Furthermore, the probabilistic spike sampling of the error ensures that the entire error region is accurately represented over trials with a constrained CF activity below 10 spikes per second, per fibre (CF activated between 1–10 Hz). Hence, the error evolution is accurately sampled even at a low frequency [148, 150]. This firing behaviour is consistent to those observed in neurophysiological recordings [149].(PDF)

## References

[1] Ito M. Cerebellar control of the VOR; around the flocculus hypothesis. Annu Rev Neurosci. 1982;5(1):275–97. 6803651 10.1146/annurev.ne.05.030182.001423

[2] Clopath C, Badura A, De Zeeuw CI, Brunel N. A cerebellar learning model of VOR adaptation in wild-type and mutant mice. J Neurosci. 2014;34(21):7203–15. 10.1523/JNEUROSCI.2791-13.2014 24849355 PMC6608186

[3] Herzfeld DJ, Kojima Y, Soetedjo R, Shadmehr R. Encoding of action by the Purkinje cells of the cerebellum. Nature. 2015;526(7573):439–42. 10.1038/nature15693 26469054 PMC4859153

[4] Lorente de Nó R. Vestibulo-ocular reflex arc. Archiv Neurol & Psychiatry. 1933.

[5] Cohen B. The VOR Arc In: Kornhuber HH, editor. Vestibular System Part 1: Basic Mechanisms: Springer Berlin Heidelberg; 1974 p. 477–540.

[6] Ito M. Error Detection and Representation in the Olivo-Cerebellar System. Front Neural Circuits. 2013:1–8. 10.3389/fncir.2013.00001 23440175 PMC3579189

[7] Sargolzaei A, Abdelghani M, Yen KK, Sargolzaei S. Sensorimotor control: computing the immediate future from the delayed present. BMC bioinformatics. 2016;17(7):245. 27454449 10.1186/s12859-016-1098-2 PMC4965729

[8] Leigh RJ, Zee DS. The neurology of eye movements: Oxford University Press; 2015.

[9] Medina JF, Lisberger SG. Links from complex spikes to local plasticity and motor learning in the cerebellum of awake-behaving monkeys. Nat Neurosci. 2008;11(10):1185–92. 10.1038/nn.2197 18806784 PMC2577564

[10] Welsh JP, Lang EJ, Suglhara I, Llinas R. Dynamic organization of motor control within the olivocerebellar system. Nature. 1995;374(6521):453–7. 10.1038/374453a0 7700354

[11] Thach WT Jr., Somatosensory receptive fields of single units in cat cerebellar cortex. J Neurophysiol. 1967;30(4):675–96. 10.1152/jn.1967.30.4.675 6035687

[12] Raman IM, Bean BP. Ionic currents underlying spontaneous action potentials in isolated cerebellar Purkinje neurons. J Neurosci. 1999;19(5):1663–74. 10024353 10.1523/JNEUROSCI.19-05-01663.1999 PMC6782167

[13] Schmolesky MT, Weber JT, Zeeuw CI, Hansel C. The making of a complex spike: ionic composition and plasticity. Ann N Y Acad Sci 2002;978(1):359–90. 12582067 10.1111/j.1749-6632.2002.tb07581.x

[14] Eccles JC, Llinás R, Sasaki K. The excitatory synaptic action of climbing fibres on the Purkinje cells of the cerebellum. J Physiol. 1966;182(2):268–96. 5944665 10.1113/jphysiol.1966.sp007824 PMC1357472

[15] Najafi F, Medina JF. Beyond "all-or-nothing" climbing fibers: graded representation of teaching signals in Purkinje cells. Front Neural Circuits. 2013;7:1–15. 10.3389/fncir.2013.00001 23847473 PMC3698456

[16] Mathy A, Ho SS, Davie JT, Duguid IC, Clark BA, Hausser M. Encoding of oscillations by axonal bursts in inferior olive neurons. Neuron. 2009;62(3):388–99. 10.1016/j.neuron.2009.03.023 19447094 PMC2777250

[17] Davie JT, Clark BA, Häusser M. The origin of the complex spike in cerebellar Purkinje cells. J Neurosci. 2008;28(30):7599–609. 10.1523/JNEUROSCI.0559-08.2008 18650337 PMC2730632

[18] Zang Y, Dieudonné S, De Schutter E. Voltage-and Branch-Specific Climbing Fiber Responses in Purkinje Cells. Cell reports. 2018;24(6):1536–49. 10.1016/j.celrep.2018.07.011 30089264

[19] Najafi F, Giovannucci A, Wang SS-H, Medina JF. Coding of stimulus strength via analog calcium signals in Purkinje cell dendrites of awake mice. eLife. 2014;3 10.7554/eLife.03663 25205669 PMC4158287

[20] Miles FA, Lisberger SG. Plasticity in the vestibulo-ocular reflex: a new hypothesis. Annual rev Neurosci. 1981;4(1):273–99. 6784658 10.1146/annurev.ne.04.030181.001421

[21] McElvain LE, Bagnall MW, Sakatos A, du Lac S. Bidirectional plasticity gated by hyperpolarization controls the gain of postsynaptic firing responses at central vestibular nerve synapses. Neuron. 2010;68(4):763–75. 10.1016/j.neuron.2010.09.025 21092864 PMC3189222

[22] Menzies JR, Porrill J, Dutia M, Dean P. Synaptic plasticity in medial vestibular nucleus neurons: comparison with computational requirements of VOR adaptation. PloS one. 2010;5(10):e13182 10.1371/journal.pone.0013182 20957149 PMC2950150

[23] Aizenman C, Manis P, Linden D. Polarity of long-term synaptic gain change is related to postsynaptic spike Neuron. 1998;21(4):827–35. 9808468 10.1016/s0896-6273(00)80598-x

[24] Morishita W, Sastry B. Postsynaptic mechanisms underlying long-term depression of gabaergic transmission in neurons of the deep cerebellar nuclei. J Neurophysiol. 1996;76(1):59–68. 10.1152/jn.1996.76.1.59 8836209

[25] Ouardouz M, Sastry B. Mechanisms underlying ltp of inhibitory synaptic transmission in the deep cerebellar nuclei. J Neurophysiol 2000 84(3):1414–21. 10.1152/jn.2000.84.3.1414 10980014

[26] Masuda N, Amari S. A computational study of synaptic mechanisms of partial memory transfer in cerebellar VOR learning. J Comput Neurosci. 2008;24(2):137–56. 10.1007/s10827-007-0045-7 17616795

[27] Badura A, Schonewille M, Voges K, Galliano E, Renier N, Gao Z, et al Climbing Fiber Input Shapes Reciprocity of Purkinje Cell Firing. Neuron. 2013;78(4):700–13. 10.1016/j.neuron.2013.03.018 23643935

[28] Najafi F. Trial-by-trial coding of instructive signals in the cerebellum: Insights from eyeblink conditioning in mice: University of Pennsylvania; 2014.

[29] Gao Z, vanBeugen BJ, De Zeeuw CI. Distributed Synergistic Plasticity and Cerebellar Learning. Nat Rev Neurosci. 2012;13:1–17. 10.1038/nrn3312 22895474

[30] Hansel C, Linden DJ, D'Angelo E. Beyond parallel fiber LTD: the diversity of synaptic and non-synaptic plasticity in the cerebellum. Nat Neurosci. 2001;4(5):467–75. 10.1038/87419 11319554

[31] Garrido JA, Luque NR, D'Angelo E, Ros E. Distributed cerebellar plasticity implements adaptable gain control in a manipulation task: a closed-loop robotic simulation. Front Neural Circuits. 2013;7. 10.3389/fncir.2013.00159 PMC3793577 24130518

[32] Luque NR, Garrido JA, Carrillo RR, D’Angelo E, Ros E. Fast convergence of learning requires plasticity between inferior olive and deep cerebellar nuclei in a manipulation task: a closed-loop robotic simulation. Front Comput Neurosci. 2014;8. 10.3389/fncom.2014.00097 PMC4133770 25177290

[33] Medina JF, Mauk MD. Computer simulation of cerebellar information processing. Nat Neurosci. 2000;3 1205–11. 10.1038/81486 11127839

[34] D’Angelo E, Mapelli L, Casellato C, Garrido JA, Luque NR, Monaco J, et al Distributed Circuit Plasticity: New Clues for the Cerebellar Mechanisms of Learning. Cerebellum. 2015:1–13. 10.1007/s12311-014-0614-z 26304953

[35] Shadmehr R, Brashers-Krug T. Functional stages in the formation of human long-term motor memory. J Neurosci. 1997;17(1):409–19. 8987766 10.1523/JNEUROSCI.17-01-00409.1997 PMC6793707

[36] Shadmehr R, Holcomb HH. Neural correlates of motor memory consolidation. Science. 1997;277(5327):821–5. 9242612 10.1126/science.277.5327.821

[37] Ohyama T, Nores WL, Medina JF, Riusech FA, Mauk MD. Learning-induced plasticity in deep cerebellar nucleus. J Neurosci. 2006;26(49):12656–63. 10.1523/JNEUROSCI.4023-06.2006 17151268 PMC6674844

[38] Kassardjian CD, Tan YF, Chung JY, Heskin R, Peterson MJ, Broussard DM. The site of a motor memory shifts with consolidation. J Neurosci. 2005;25(35):7979–85. 10.1523/JNEUROSCI.2215-05.2005 16135754 PMC6725450

[39] Anzai M, Kitazawa H, Nagao S. Effects of reversible pharmacological shutdown of cerebellar flocculus on the memory of long-term horizontal VOR adaptation in monkeys. Neurosci Res. 2010;68(3):191–8. 10.1016/j.neures.2010.07.2038 20674618

[40] Van Alphen AM, Stahl JS, De Zeeuw CI. The dynamic characteristics of the mouse horizontal vestibulo-ocular and optokinetic response. Brain Res. 2001;890(2):296–305. 11164796 10.1016/s0006-8993(00)03180-2

[41] McKay BE, Engbers JDT, Mehaffey WH, Gordon GRJ, Molineux ML, Bains JS, et al Climbing Fiber Discharge Regulates Cerebellar Functions by Controlling the Intrinsic Characteristics of Purkinje Cell Output. J Neurophysiol. 2007;97(4):2590–604. 10.1152/jn.00627.2006 17267759

[42] Llinás R, Sugimori M. Electrophysiological properties of in vitro Purkinje cell somata in mammalian cerebellar slices. J Physiol. 1980;305:171–95. 7441552 10.1113/jphysiol.1980.sp013357 PMC1282966

[43] Llinás R, Sugimori M. Electrophysiological properties of in vitro Purkinje cell dendrites in mammalian cerebellar slices. J Physiol. 1980;305:197–213. 7441553 10.1113/jphysiol.1980.sp013358 PMC1282967

[44] Grasselli G, He Q, Wan V, Adelman JP, Ohtsuki G, Hansel C. Activity-Dependent Plasticity of Spike Pauses in Cerebellar Purkinje Cells. Cell Reports. 2016;14(11):2546–53. 10.1016/j.celrep.2016.02.054 26972012 PMC4805497

[45] Minor LB, Goldberg JM. Vestibular-nerve inputs to the VOR a functional-ablation study in the squirrel monkey. J Neurosci. 1991;11(6):1636–48. 2045879 10.1523/JNEUROSCI.11-06-01636.1991 PMC6575423

[46] Williams JA, Bridgeman B, Woods T, Welch R. Global VOR gain adaptation during near fixation to foveal targets. Hum Mov Sci. 2007;26(6):787–95. 10.1016/j.humov.2007.06.002 17870197

[47] Gonshor A, Jones GM. Extreme vestibulo‐ocular adaptation induced by prolonged optical reversal of vision. J Physiol. 1976;256(2):381–414. 16992508 10.1113/jphysiol.1976.sp011330 PMC1309313

[48] Mano N. Changes of simple and complex spike activity of cerebellar Purkinje cells with sleep and waking. Science. 1970;170(3964):1325–7. 4320259 10.1126/science.170.3964.1325

[49] Marchesi GF, Strata P. Mossy and climbing fiber activity during phasic and tonic phenomena of sleep. Pflügers Archiv. 1971;323(3):219–40. 4322916 10.1007/BF00586385

[50] Luebke AE, Robinson DA. Gain changes of the cat's VOR after flocculus deactivation. Exp Brain Res. 1994;98(3):379–90. 8056061 10.1007/BF00233976

[51] Albus JS. A theory of cerebellar function. Math Biosci. 1971;10:25–61.

[52] Marr D. A theory of cerebellar cortex. J Physiol. 1969;202:437–70. 5784296 10.1113/jphysiol.1969.sp008820 PMC1351491

[53] Howell FW, Dyhrfjeld-Johnsen J, Maex R, Goddard N, Schutter ED. A large-scale model of the cereb. cortex using PGENESIS. Neurocomp. 2000;32–3:1041–46.

[54] Maex R, De Schutter E. Synchronization of Golgi and granule cell firing in a detailed network model of the cerebellar granule cell layer. J Neurophysiol. 1998;80(5):2521–37. 10.1152/jn.1998.80.5.2521 9819260

[55] Schweighofer N. Computational Models of the Cerebellum in the Adaptive Control of Movements. PhD thesis. 1995.

[56] Schweighofer N, Arbib MA, Kawato M. Role of the cerebellum in reaching movements in human. I. Distributed Inverse dynamics control. Eur J Neurosci 1998;10:86–94. 9753116 10.1046/j.1460-9568.1998.00006.x

[57] Solinas S, Nieus T, D'Angelo E. A realistic large-scale model of the cerebellum granular layer predicts circuit spatio temporal filtering properties. Front Cell Neurosci. 2010;4(0). 10.3389/fncel.2010.00012 PMC2876868 20508743

[58] Tolu S, Vanegas M, Garrido JA, Luque NR, Ros E. Adaptive and Predictive Control of a Simulated Robot Arm. Int J Neural Syst. 2013;23(3). 10.1142/S012906571350010X 23627657

[59] Fujita M. Adaptive filter model of the cerebellum. Biol Cybern. 1982;45(3):195–206. 7171642 10.1007/BF00336192

[60] Porrill J, Dean P. Cerebellar Motor Learning: When Is Cortical Plasticity Not Enough? PLOS Comput Biol. 2007;3(10). 10.1371/journal.pcbi.0030197 PMC2041974 17967048

[61] Tolu S, Vanegas M, Luque NR, Garrido JA, Ros E. Bio-inspired Adaptive FEL Architecture for Motor Control. Biol Cybern. 2012;106(8–9):507–22. 10.1007/s00422-012-0515-5 22907270

[62] Blazquez PM, Hirata Y, Heiney SA, Green AM, Highstein SM. Cerebellar signatures of VOR motor learning. J Neurosci. 2003;23(30):9742–51. 14586001 10.1523/JNEUROSCI.23-30-09742.2003 PMC6740887

[63] De Zeeuw CI, Simpson JI, Hoogenraad CC, Galjart N, Koekkoek SKE, J. R. Microcircuitry and function of the inferior olive. Trends Neurosci. 1998;21(9):391–400. 9735947 10.1016/s0166-2236(98)01310-1

[64] Rambold H, Churchland A, Selig Y, Jasmin L, Lisberger S. Partial ablations of the flocculus and ventral paraflocculus in monkeys cause linked deficits in smooth pursuit eye movements and adaptive modification of the VOR. J Neurophysiol. 2002;87(2):912–24. 10.1152/jn.00768.2000 11826056 PMC2629758

[65] Kawato M. Feedback-error-learning neural network for supervised motor learning. Advanced neural computers. 1990;6(3):365–72.

[66] Franchi E, Falotico E, Zambrano D, Muscolo GG, Marazzato L, Dario P, et al., editors. A comparison between two bio-inspired adaptive models of Vestibulo-Ocular Reflex (VOR) implemented on the iCub robot. Humanoid Robots (Humanoids), 2010 10th IEEE-RAS International Conference on; 2010: IEEE.

[67] Shibata T, Schaal S. Biomimetic gaze stabilization based on feedback-error-learning with nonparametric regression networks. Neural Networks. 2001;14(2):201–16. 11316234 10.1016/s0893-6080(00)00084-8

[68] Lisberger S, Sejnowski T. Motor learning in a recurrent network model based on the vestibulo–ocular reflex. Nature. 1992;360(6400):159 10.1038/360159a0 1436091

[69] Porrill J, Dean P, Stone JV. Recurrent cerebellar architecture solves the motor-error problem. Proceedings of the Royal Society of London-B. 2004;271(1541):789–96. 10.1098/rspb.2003.2658 PMC1691672 15255096

[70] Dean P, Porrill J, Stone JV. Decorrelation control by the cerebellum achieves oculomotor plant compensation in simulated vestibulo-ocular reflex. Proceedings of the Royal Society of London B: Biological Sciences. 2002;269(1503):1895–904. 10.1098/rspb.2002.2103 PMC1691115 12350251

[71] Vijayakumar S, Schaal S, editors. Locally weighted projection regression: An O (n) algorithm for incremental real time learning in high dimensional space. Proceedings of the Seventeenth International Conference on Machine Learning (ICML 2000); 2000.

[72] Vannucci L, Tolu S, Falotico E, Dario P, Lund HH, Laschi C, editors. Adaptive gaze stabilization through cerebellar internal models in a humanoid robot. Biomedical Robotics and Biomechatronics (BioRob), 2016 6th IEEE International Conference on; 2016: IEEE.

[73] Badura A, Clopath C, Schonewille M, De Zeeuw CI. Modeled changes of cerebellar activity in mutant mice are predictive of their learning impairments. Sci Rep. 2016;6:36131 10.1038/srep36131 27805050 PMC5095348

[74] Yamazaki T, Nagao S, Lennon W, Tanaka S. Modeling memory consolidation during posttraining periods in cerebellovestibular learning. PNAS. 2015;112(11):3541–46. 10.1073/pnas.1413798112 25737547 PMC4371920

[75] Latorre R, Aguirre C, Rabinovich MI, Varona P. Transient dynamics and rhythm coordination of inferior olive spatio-temporal patterns. Front Neural Circuits. 2013;7(138):1–18. 10.3389/fncir.2013.00138 24046731 PMC3763220

[76] Schweighofer N, Doya K, Kawato M. Electrophysiological properties of inferior olive neurons: a compartmental model. J Neurophysiol. 1999;82(2):804–17. 10.1152/jn.1999.82.2.804 10444678

[77] De Gruijl JR, Bazzigaluppi P, de Jeu MTG, De Zeeuw CI. Climbing Fiber Burst Size and Olivary Sub-threshold Oscillations in a Network Setting. PLOS Comput Biol. 2012;8(12). 10.1371/journal.pcbi.1002814 PMC3521668 23271962

[78] Middleton SJ, Racca C, Cunningham MO, Traub RD, Monyer H, Knopfel T, et al High-frequency network oscillations in cerebellar cortex. Neuron. 2008;58(5):763–74. 10.1016/j.neuron.2008.03.030 18549787 PMC4852717

[79] Miyasho T, Takagi H, Suzuki H, Watanabe S, Inoue M, Kudo Y, et al Low-threshold potassium channels and a low-threshold calcium channel regulate Ca2+ spike firing in the dendrites of cerebellar Purkinje neurons: a modeling study. Brain Res. 2001;891(1–2):106–15. 11164813 10.1016/s0006-8993(00)03206-6

[80] Kimpo RR, Boyden ES, Katoh A, Ke MC, Raymond JL. Distinct patterns of stimulus generalization of increases and decreases in VOR gain. J Neurophysiol. 2005;94(5):3092–100. 10.1152/jn.00048.2005 16033945

[81] Kimpo RR, Rinaldi JM, Kim CK, Payne HL, Raymond JL. Gating of neural error signals during motor learning. Elife. 2014;3:e02076 10.7554/eLife.02076 24755290 PMC3989583

[82] Stone LS, Lisberger SG. Visual responses of Purkinje cells in the cerebellar flocculus during smooth-pursuit eye movements in monkeys. I. Simple spikes. J Neurophysiol. 1990;63(5):1241–61. 10.1152/jn.1990.63.5.1241 2358872

[83] Boyden ES, Katoh A, Raymond JL. Cerebellum-Dependent Learning: The Role of Multiple Plasticity Mechanisms. Annu Rev Neurosci. 2004;27(1):581–609. 15217344 10.1146/annurev.neuro.27.070203.144238

[84] Boucheny C, Carrillo RR, Ros E, Coenen OJ-MD. Real-time spiking neural network: an adaptive cerebellar model. LNCS. 2005;3512:136–44.

[85] Kettner RE, Mahamud S, Leung H, Sittko N, Houk JC, Peterson BW, et al Prediction of complex two-dimensional trajectories by a cerebellar model of smooth pursuit eye movement. J Neurophysiol. 1997;77(4):2115–30. 10.1152/jn.1997.77.4.2115 9114259

[86] Ros E, Carrillo RR, Ortigosa EM, Barbour B, Agís R. Event-driven simulation scheme for spiking neural networks using lookup tables to characterize neuronal dynamics. Neural Comput. 2006;18(12):2959–93. 10.1162/neco.2006.18.12.2959 17052155

[87] Belmeguenai A, Botta P, Weber JT, Carta M, De Ruiter M, De Zeeuw CI, et al Alcohol Impairs LTD at the Cerebellar Parallel Fiber–Purkinje Cell Synapse. J Neurophysiol. 2008;100(6):3167–74. 10.1152/jn.90384.2008 18922952 PMC2604851

[88] He Q, Titley H, Grasselli G, Piochon C, Hansel C. Ethanol affects NMDA receptor signaling at climbing fiber-Purkinje cell synapses in mice and impairs cerebellar LTD. J Neurophysiol. 2013;109(5):1333–42. 10.1152/jn.00350.2012 23221414 PMC3602830

[89] Carey MR, Regehr WG. Noradrenergic Control of Associative Synaptic Plasticity by Selective Modulation of Instructive Signals. Neuron. 2009;62(1):112–22. 10.1016/j.neuron.2009.02.022 19376071 PMC2837271

[90] Ke MC, Guo CC, Raymond JL. Elimination of climbing fiber instructive signals during motor learning. Nat Neurosci. 2009;12(9):1171–9. 10.1038/nn.2366 19684593 PMC3864777

[91] Popa LS, Streng ML, Hewitt AL, Ebner TJ. The Errors of Our Ways: Understanding Error Representations in Cerebellar-Dependent Motor Learning. Cerebellum. 2015. 10.1007/s12311-015-0685-5 PMC4691440 26112422

[92] Ohmae S, Medina JF. Climbing fibers encode a temporal-difference prediction error during cerebellar learning in mice. Nat Neurosci. 2015;18(12):1798–803. 10.1038/nn.4167 26551541 PMC4754078

[93] Schonewille M, Gao Z, Boele H-J, Veloz MFV, Amerika WE, Šimek AA, et al Reevaluating the role of LTD in cerebellar motor learning. Neuron. 2011;70(1):43–50. 10.1016/j.neuron.2011.02.044 21482355 PMC3104468

[94] Bengtsson F, Hesslow G. Cerebellar control of the inferior olive. Cerebellum. 2006;review article:1–8. 10.1080/14734220500462757 16527758

[95] Welberg L. Cerebellum: An olive branch to two theories. Nat Rev Neurosci. 2009;10:468.

[96] Kawato M, Gomi H. A computational model of four regions of the cerebellum based on FEL. Biol Cybern. 1992;68(2):95–103. 1486143 10.1007/BF00201431

[97] Bazzigaluppi P, R. DGJ, Van Der Giessen RS, Khosrovani S, De Zeeuw CI, De Jeu MTG. Olivary subthreshold oscillations and burst activity revisited Front Neural Circuits. 2012;6(91). 10.3389/fncir.2012.00091 PMC3504313 23189043

[98] Maruta J, Hensbroek RA, Simpson JI. Intraburst and interburst signaling by climbing fibers. J Neurosci. 2007;27(42):11263–70. 10.1523/JNEUROSCI.2559-07.2007 17942720 PMC6673016

[99] Llinas R, Welsh JP. On the cerebellum and motor learning. Curr Opin Neurobiol. 1993;3:958–65. 8124080 10.1016/0959-4388(93)90168-x

[100] Placantonakis DG, Bukovsky AA, Zeng X-H, Kiem H-P, Welsh JP. Fundamental role of inferior olive connexin 36 in muscle coherence during tremor. PNAS. 2004;101(18):7164–9. 10.1073/pnas.0400322101 15103021 PMC406483

[101] Keating JG, Thach WT. Nonclock behavior of inferior olive neurons. Interspike interval of Purkinje cell complex spike discharge in the awake behaving monkey is random. J Neurophysiol. 1995;73(4):1329–40. 10.1152/jn.1995.73.4.1329 7643151

[102] Xu D, Liu T, Ashe J, Bushara KO. Role of the olivo-cerebellar system in timing. J Neurosci. 2006;26(22):5990–95. 10.1523/JNEUROSCI.0038-06.2006 16738241 PMC6675226

[103] Liu T, Xu D, Ashe J, Bushara K. Specificity of inferior olive response to stimulus timing. J Neurophysiol. 2008;100(3):1557–61. 10.1152/jn.00961.2007 18632890 PMC2544464

[104] Wu X, Ashe J, Bushara KO. Role of olivocerebellar system in timing without awareness. PNAS. 2011;108(33):13818–22. 10.1073/pnas.1104096108 21808015 PMC3158188

[105] Gibson AR, Horn KM, Pong M. Activation of climbing fibers. Cerebellum. 2004;3(4):212–21. 10.1080/14734220410018995 15686099

[106] Kitazawa S, Wolpert DM. Rhythmicity, randomness and synchrony in climbing fiber signals. Trends Neurosci. 2005;28(11):611–9. 10.1016/j.tins.2005.09.004 16182386

[107] Llinás R, Welsh JP. On the cerebellum and motor learning. Current opinion in neurobiology. 1993;3(6):958–65. 8124080 10.1016/0959-4388(93)90168-x

[108] Llinás R. Inferior olive oscillation as the temporal basis for motricity and oscillatory reset as the basis for motor error correction. Neurosci. 2009;162(3):797–804. 10.1016/j.neuroscience.2009.04.045 PMC2861300 19393291

[109] De Zeeuw CI, Hoogenraad CC, Koekkoek SKE, Ruigrok TJ, Galjart N, Simpson JI. Microcircuitry and function of the inferior olive. Trends Neurosci. 1998;21(9):391–400. 9735947 10.1016/s0166-2236(98)01310-1

[110] Steuber V, Mittmann W, Hoebeek FE, Silver RA, De Zeeuw CI, Häusser M, et al Cerebellar LTD and pattern recognition by Purkinje cells. Neuron. 2007;54(1):121–36. 10.1016/j.neuron.2007.03.015 17408582 PMC1885969

[111] Zhou H, Voges K, Lin Z, Ju C, Schonewille M. Differential Purkinje cell simple spike activity and pausing behavior related to cerebellar modules. J Neurophysiol. 2015;113(7):2524–36. 10.1152/jn.00925.2014 25717166 PMC4416590

[112] Schonewille M, Khosrovani S, Winkelman BH, Hoebeek FE, De Jeu MT, Larsen IM, et al Purkinje cells in awake behaving animals operate at the upstate membrane potential. Nat Neurosci. 2006;9(4):459–61;. 10.1038/nn0406-459 16568098

[113] Mori K. Across-frequency nonlinear inhibition by GABA in processing of interaural time difference. Hearing research. 1997;111(1–2):22–30. 9307308 10.1016/s0378-5955(97)00090-7

[114] Bower JM. Model-founded explorations of the roles of molecular layer inhibition in regulating purkinje cell responses in cerebellar cortex: more trouble for the beam hypothesis. Front Cellular Neurosci. 2010;4:27. 10.3389/fncel.2010.00027 PMC2944648 20877427

[115] Wisden W, Murray AJ, McClure CJ, Wulff P. Studying cerebellar circuits by remote control of selected neuronal types with GABA-A receptors. Front Mol Neurosci. 2009;2:29 10.3389/neuro.02.029.2009 20076763 PMC2805427

[116] Wulff P, Schonewille M, Renzi M, Viltono L, Sassoè-Pognetto M, Badura A, et al Synaptic inhibition of Purkinje cells mediates consolidation of vestibulo-cerebellar motor learning. Nat Neurosci. 2009;12(8):1042 10.1038/nn.2348 19578381 PMC2718327

[117] Santamaria F, Tripp PG, Bower JM. Feedforward inhibition controls the spread of granule cell–induced Purkinje cell activity in the cerebellar cortex. J Neurophysiol. 2007;97(1):248–63. 10.1152/jn.01098.2005 17050824

[118] Korbo L, Andersen BB, Ladefoged O, Møller A. Total numbers of various cell types in rat cerebellar cortex estimated using an unbiased stereological method. Brain research. 1993;609(1–2):262–8. 8508308 10.1016/0006-8993(93)90881-m

[119] Friedel P, van Hemmen JL. Inhibition, not excitation, is the key to multimodal sensory integration. Biol Cybern. 2008;98(6):597–618. 10.1007/s00422-008-0236-y 18491169

[120] Luque NR, Garrido JA, Carrillo RR, Coenen OJMD, Ros E. Cerebellar Input Configuration Toward Object Model Abstraction in Manipulation Tasks. IEEE Trans Neural Netw. 2011;22(8):1321–8. 10.1109/TNN.2011.2156809 21708499

[121] Canto CB, Onuki Yi, Bruinsma B, van der Werf YD, De Zeeuw CI. The Sleeping Cerebellum. Trends Neurosci. 2017. 10.1016/j.tins.2017.03.001 28431742

[122] Eccles JC, Ito M, Szentágothai J. The Cerebellum as a Neuronal Machine New York: Springer-Verlag; 1967.

[123] Ito M. The cerebellum and neural control. 1984.

[124] Medina J, Mauk M. Simulations of cerebellar motor learning: computational analysis of plasticity at the mossy fiber synapse. J Neurosci. 1999;19(16):7140–51. 10436067 10.1523/JNEUROSCI.19-16-07140.1999 PMC6782874

[125] Voogd J, Glickstein M. The anatomy of the cerebellum. Trends Neurosci. 1998;21(9):370–5. 9735944 10.1016/s0166-2236(98)01318-6

[126] Luque NR, Garrido JA, Naveros F, Carrillo RR, D‘Angelo E, Ros E. Distributed Cerebellar Motor Learning; a STDP Model. Front Comp Neurosci. 2016;10 10.3389/fncom.2016.00017 26973504 PMC4773604

[127] Naveros F, Luque NR, Garrido JA, Carrillo RR, Anguita M, Ros E. A Spiking Neural Simulator Integrating Event-Driven and Time-Driven Computation Schemes Using Parallel CPU-GPU Co-Processing: A Case Study. IEEE Trans Neural Netw Learn Syst. 2015;26(7):1567–74. 10.1109/TNNLS.2014.2345844 25167556

[128] Naveros F, Garrido JA, Carrillo RR, Ros E, Luque NR. Event-and Time-Driven Techniques Using Parallel CPU-GPU Co-processing for Spiking Neural Networks. Front Neuroinformatics. 2017;11. 10.3389/fninf.2017.00007 PMC5293783 28223930

[129] Bezzi M, Nieus T, Coenen OJ-MD, D'Angelo E. An I&F model of a cerebellar granule cell. Neurocomp. 2004;58:593–8.

[130] Gerstner W, Kistler WM. Spiking neuron models: Single neurons, populations, plasticity: Cambridge university press; 2002.

[131] Hurlock EC, McMahon A, Joho RH. Purkinje-cell-restricted restoration of Kv3. 3 function restores complex spikes and rescues motor coordination in Kcnc3 mutants. J Neurosci. 2008;28(18):4640–8. 10.1523/JNEUROSCI.5486-07.2008 18448641 PMC6670432

[132] Vigot R, Batini C. GABAB receptor activation of Purkinje cells in cerebellar slices. Neuroscience research. 1997;29(2):151–60. 9359464 10.1016/s0168-0102(97)00087-4

[133] Silver RA, Colquhoun D, CullCandy SG, Edmonds B. Deactivation and desensitization of non-NMDA receptors in patches and the time course of EPSCs in rat cerebellar granule cells. J Physiol. 1996;493:167–73. 8735702 10.1113/jphysiol.1996.sp021372 PMC1158958

[134] Tia S, Wang JF, Kotchabhakdi N, Vicini S. Developmental changes of inhibitory synaptic currents in cerebellar granule neurons: Role of GABA(A) receptor alpha 6 subunit. J Neurosci. 1996;16:3630–40. 8642407 10.1523/JNEUROSCI.16-11-03630.1996 PMC6578841

[135] Nusser Z, CullCandy S, Farrant M. Differences in synaptic GABA(A) receptor number underlie variation in GABA mini amplitude. Neuron. 1997;19:697–709. 9331359 10.1016/s0896-6273(00)80382-7

[136] Rossi DJ, Hamann M. Spillover-mediated transmission at inhibitory synapses promoted by high affinity alpha(6) subunit GABA(A) receptors and glomerular geometry. Neuron. 1998;20:783–95. 9581769 10.1016/s0896-6273(00)81016-8

[137] D’Angelo E, Defilippi G, Rossi P, Taglietti V. Ionic mechanism of electroresponsiveness in cerebellar granule cells implicates the action of a persistent sodium current. J Neurophysiol. 1998;80:493–503. 10.1152/jn.1998.80.2.493 9705445

[138] DiGregorio DA, Nusser Z, Silver RA. Spillover of glutamate onto synaptic AMPA receptors enhances fast transmission at a cerebellar synapse. Neuron. 2002;35(3):521–33. 12165473 10.1016/s0896-6273(02)00787-0

[139] D’Angelo E, Nieus T, Maffei A, Armano S, Rossi P. Theta-frequency bursting and resonance in cerebellar granule cells: experimental evidence and modeling of a slow K+-dependent mechanism. J Neurosci. 2001;21:759–70. 11157062 10.1523/JNEUROSCI.21-03-00759.2001 PMC6762330

[140] D’Angelo E, Rossi P, Taglietti V. Different proportions of N-Methyl-D-Aspartate and Non-N-Methyl-D-Aspartate receptor currents at the mossy fiber granule cell synapse of developing rat cerebellum. Neuroscience. 1993;53:121–30. 8097019 10.1016/0306-4522(93)90290-v

[141] Nieus T, Sola E, Mapelli J, Saftenku E, Rossi P. LTP regulates burst initiation and frequency at mossy fiber-granule cell synapses of rat cerebellum: Experimental observations and theoretical predictions. Journal of Neurophysiology. 2006;95:686–99. 10.1152/jn.00696.2005 16207782

[142] Lisberger SG, Fuchs AF. Role of primate flocculus during rapid behavioral modification of VOR. II. Mossy fiber firing patterns during horizontal head rotation and eye movement. J Neurophysiol. 1978;41(3):764–77. 10.1152/jn.1978.41.3.764 96226

[143] Arenz A, Silver RA, Schaefer AT, Margrie TW. The Contribution of Single Synapses to Sensory Representation in Vivo. Science 2008;321(5891):977–80. 10.1126/science.1158391 18703744 PMC2771362

[144] Yamazaki T, Tanaka S. The cerebellum as a liquid state machine. Neural Netw. 2007;20:290–7. 10.1016/j.neunet.2007.04.004 17517494

[145] Yamazaki T, Tanaka S. Computational models of timing mechanisms in the cerebellar granular layer. Cerebellum. 2009;8(4):423–32. 10.1007/s12311-009-0115-7 19495900 PMC2788136

[146] Yamazaki T, Tanaka S. Neural modeling of an internal clock. Neural Comput. 2005;17(5):1032–58. 10.1162/0899766053491850 15829099

[147] Honda T, Yamazaki T, Tanaka S, Nagao S, Nishino T. Stimulus-dependent state transition between synchronized oscillation and randomly repetitive burst in a model cerebellar granular layer. PLOS Comput Biol. 2011;7(7):e1002087 10.1371/journal.pcbi.1002087 21779155 PMC3136428

[148] Luque NR, Garrido JA, Carrillo RR, Coenen OJMD, Ros E. Cerebellarlike Corrective Model Inference Engine for Manipulation Tasks. IEEE Trans Syst Man Cybern. 2011;41(5):1299–312. 10.1109/TSMCB.2011.2138693 21536535

[149] Kuroda S, Yammoto K, Miyamoto H, Doya K, Kawato M. Statistical characteristics of climbing fiber spikies necessary for efficient cerebellar learning. Biol Cybern. 2001;84(3):183–92. 10.1007/s004220000206 11252636

[150] Carrillo RR, Ros E, Boucheny C, Coenen O-JM-D. A real time spiking cerebellum model for learning robto control. Biosystems. 2008;94(1–2):18–27. 10.1016/j.biosystems.2008.05.008 18616974

[151] Kano M, Hashimoto K, Kurihara H, Watanabe M, Inoue Y, Aiba A, et al Persistent Multiple Climbing Fiber Innervationof Cerebellar Purkinje Cellsin Mice Lacking mGluR1. Neuron. 1997;18(1):71–9. 10.1016/S0896-6273(01)80047-7 9010206

[152] Brunel N, Hakim V, Isope P, Nadal JP, Barbour B. Optimal information storage and the distribution of synaptic weights: perceptron versus Purkinje cell. Neuron. 2004;43:745–57. 10.1016/j.neuron.2004.08.023 15339654

[153] Schrauwen B, Van Campenhout J, editors. BSA, a fast and accurate spike train encoding scheme. Neural Netw, 2003 Proc Int Jt Conf; 2003: IEEE.

[154] Victor JD. Spike train metrics. Curr Opin Neurobiol. 2005;15(5):585–92. 10.1016/j.conb.2005.08.002 16140522 PMC2713191

[155] van Rossum MC. A novel spike distance. Neural Comput. 2001;13(4):751–63. 11255567 10.1162/089976601300014321

[156] Luque NR, Carrillo RR, Naveros F, Garrido JA, Sáez-Lara MJ. Integrated neural and robotic simulations. Simulation of cerebellar neurobiological substrate for an object-oriented dynamic model abstraction process. Rob Auton Syst. 2014;62(12):1702–16.

[157] Luque NR, Garrido JA, Carrillo RR, Tolu S, Ros E. Adaptive cerebellar spiking model embedded in the control loop: Context switching and robustness against noise. Int J Neural Syst. 2011;21(05):385–401. 21956931 10.1142/S0129065711002900

[158] Lev-Ram V, Mehta SB, Kleinfeld D, Tsien RY. Reversing cerebellar long-term depression. PNAS. 2003;100(26):15989–93. 10.1073/pnas.2636935100 14671315 PMC307680

[159] Kleberg FI, Fukai T, Gilson M. Excitatory and inhibitory STDP jointly tune feedforward neural circuits to selectively propagate correlated spiking activity. Front Comp Neurosci. 2014;8. 10.3389/fncom.2014.00053 PMC4019846 24847242

[160] Song S, Miller KD, Abbott LF. Competitive Hebbian learning through spike-timing-dependent synaptic plasticity. Nat Neurosci. 2000;3(9):919–26. 10.1038/78829 10966623

[161] Rubin J, Lee DD, Sompolinsky H. Equilibrium properties of temporally asymmetric Hebbian plasticity. Physical rev letters. 2001;86(2):364. 10.1103/PhysRevLett.86.364 11177832

[162] Demer JL, Oas JG, Baloh RW. Visual-vestibular interaction in humans during active and passive, vertical head movement. J Vestib Res. 1993;3(2):101–14. 8275247

[163] Dumas G, Perrin P, Ouedraogo E, Schmerber S. How to perform the skull vibration-induced nystagmus test (SVINT). European annals of otorhinolaryngology, head and neck diseases. 2016;133(5):343–8. 10.1016/j.anorl.2016.04.002 27161530

