# Supplementary figures and images for: Spike burst-pause dynamics of Purkinje cells regulate sensorimotor adaptation
Source: PLoS Comput Biol. 2019 Mar 12;15(3):e1006298. doi: 10.1371/journal.pcbi.1006298 (PMC6430425; doi:10.1371/journal.pcbi.1006298)

A

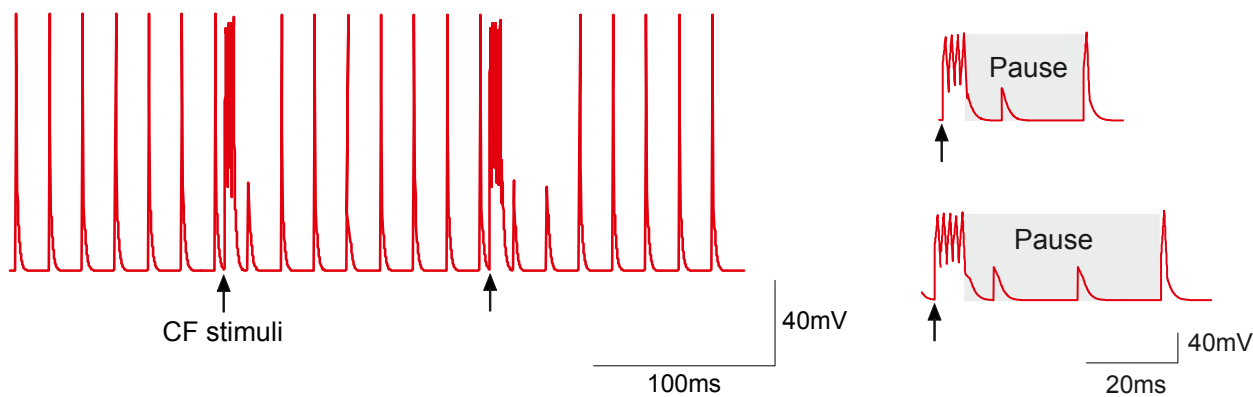

B

CS Purkinje cell

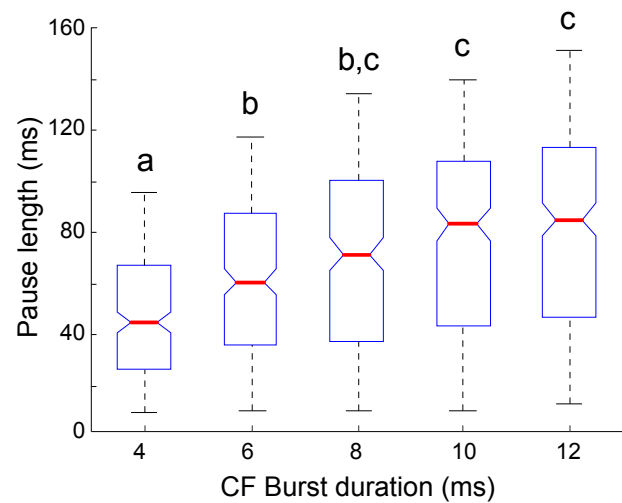

C

CS Purkinje cell  
with stochastic length pauses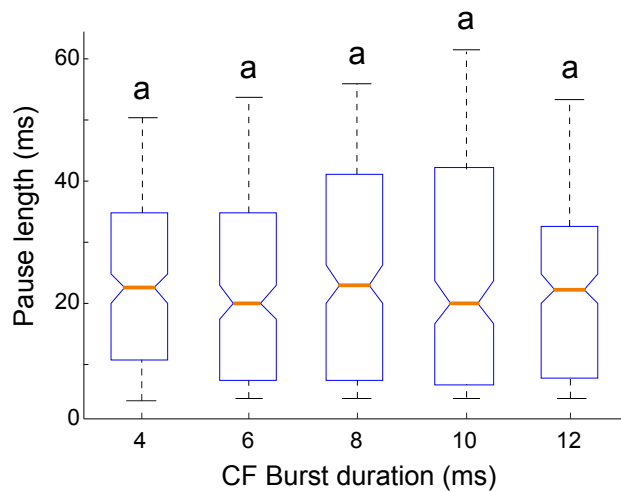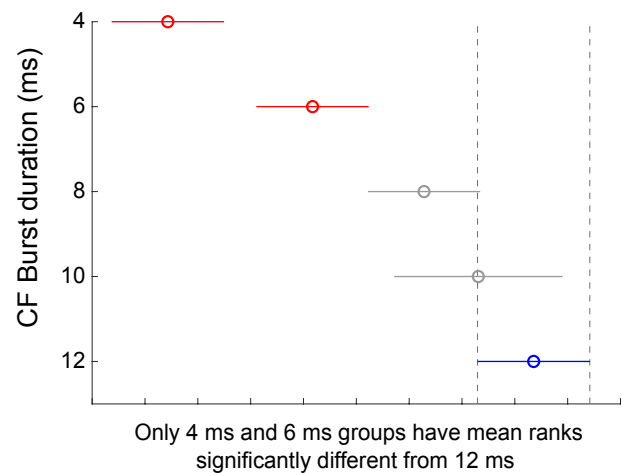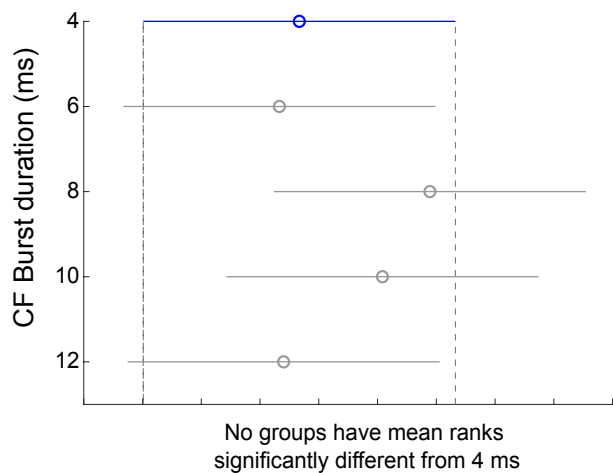

Supplement: S1 Fig — (A) In the model, CF signals modulate both the burst size (i.e., the number of spikes within the burst [17, 18]) and the duration of post-complex spike pause. (B) Across multiple simulations, we progressively increased the size of CF burst stimulation: from 4 ms (i.e., 2 spikes) to 12 ms (i.e., 6 spikes), by steps of 2 ms. For each of the 5 stimulation conditions, we varied the depolarisation current injected through PFs to elicit Purkinje responses within their operative frequency range (i.e., 50–250 Hz). We then used a Kruskal-Wallis test to assess the relationship between Purkinje spike pause lengths and CF burst duration. We found a statistically significant difference (Chi square = 145.61, p<10−20, df = 4) amongst the five conditions (i.e. CF burst sizes: 4, 6, 8, 10, and 12 ms). A Bonferroni post hoc test revealed that only the conditions 4 ms and 6 ms produced significantly shorter pauses, whereas the non-linear relation plateaued from 6–8 to 12 ms. (C) In the Purkinje cell model, the CF stimulation–CS pause length relationship is mediated by the muscarinic receptor channel. We simulated a random modulation of the time constant of the muscarinic receptor ion channel to generate stochastic Purkinje post-complex spike pauses (i.e. independently from CF stimulation). To do so, we multiplied the time constant of the muscarinic channel by a random factor at each time step (0.002 ms). Hence, whilst the activation/inactivation of the muscarinic channel remained unaltered, therefore maintaining Purkinje spike bursting, the duration of pauses was randomly modulated. The modified Purkinje cell model was used to run the same series of simulations as in B by gradually increasing the CF burst size (i.e., 4, 6, 8, 10, 12 ms). The Kruskal-Wallis test confirmed that the inserted stochastic mechanism removed any correlation between the length of Purkinje spike pauses and the CF burst sizes (Chi square = 4.06, p = 0.398, df = 4; S1C Fig). The model with random length post-comp [file pcbi.1006298.s001.pdf]

A

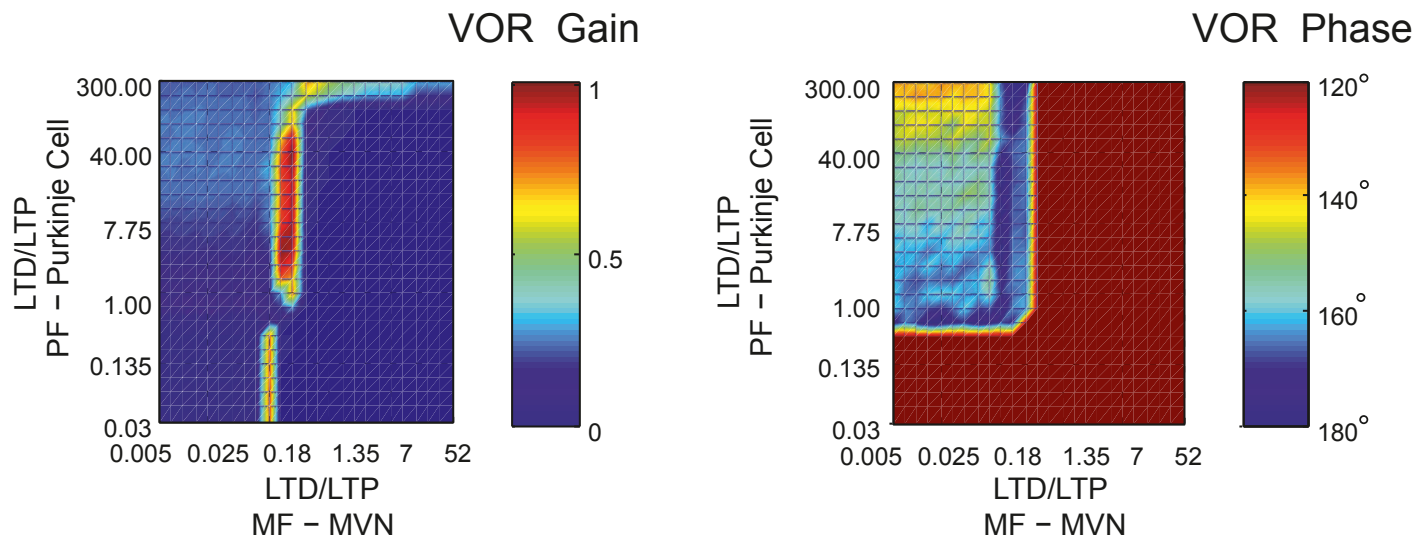

B

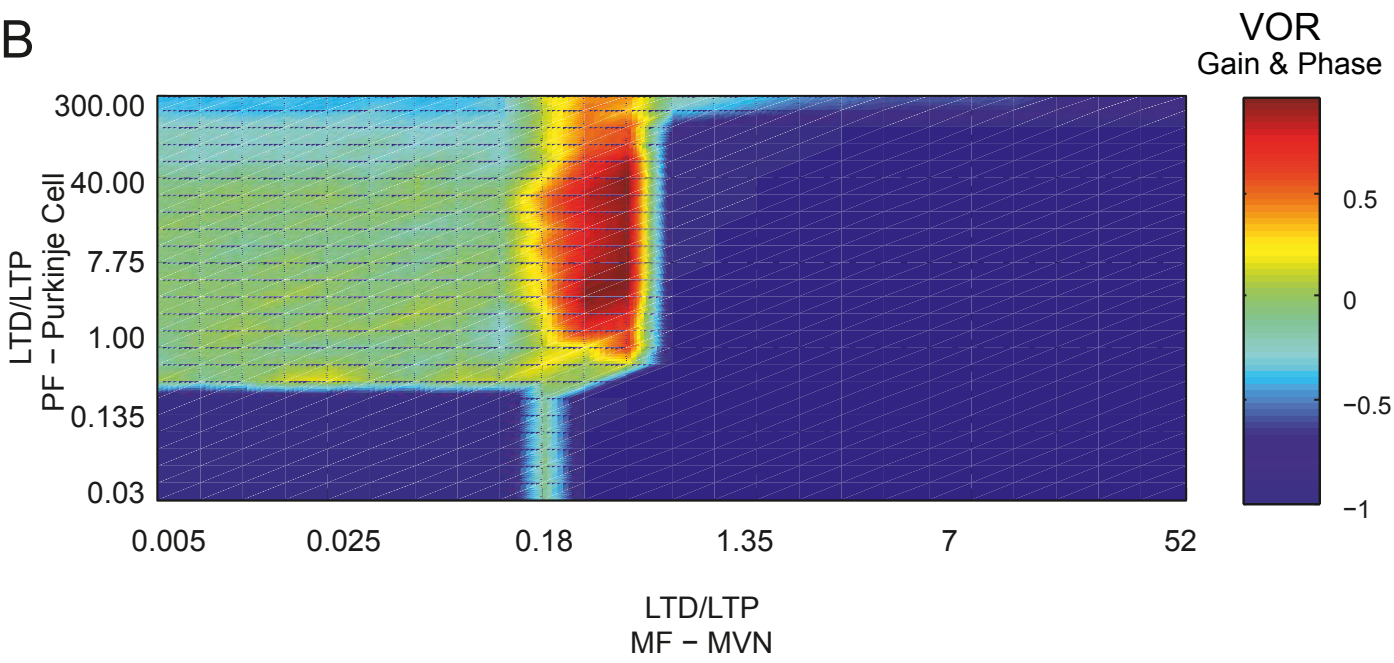

Supplement: S2 Fig — Parameter sensitivity analysis. Cerebellar adaptation modulates PF-Purkinje cell synaptic weights as well as MF-MVN synapses [6, 126]. For synaptic adaptation, the model uses supervised STDP, which exploits the interaction amongst unsupervised and supervised cell inputs to regulate and stabilise postsynaptic activity. Balancing supervised STDP, and the resulting synaptic modification dynamics, is critical, given the high sensitivity of the process that determines the LTD/LTP ratio [160, 161]. A sensitivity analysis of the parameters governing LTD and LTP, shows that LTP exceeding LTD values for a narrow range at MF-MVN synapses preserves VOR learning stability. This holds independently for both VOR gain and phase (A) as well as for the combination of the two (B). By contrast, PF-Purkinje cell synapses admit broader limits for the LTD/LTP ratio (A, B). Detailed description: we systematically simulated LTP/LTD ratio values at PF-Purkinje cell and MF-MVN synapses within a plausible range that may satisfy the expected h-VOR outcome. As simulations ran, the solutions were iteratively checked until finding the set of LTD/LTP ratio values that exhibited the better performance in terms of h-VOR gain and phase. LTD/LTP balance at each site was modified by systematically multiplying LTD by 1.5N where –11 ≤ N ≤ 12 for PF-Purkinje cell and MF-MVN synapses. For each parameter setting, the cerebellar model underwent 10 000 s of VOR learning (1Hz head rotation movement to be compensated by contralateral eye movements. (A) Final VOR gain and phase plotted over the LTD/LTP range of values that were tested. (B) Combined VOR gain and phase (normalised) as a function of the LTD/LTP ratio. At PF-Purkinje cell synapses the LTD/LTP was well balanced for N values ranging between [–1, 7]. At MF-MVN the LTD/LTP balance was more critical since N is within a narrower band range [–1, 0]. The reddish area within the last plot indicates the optimal parameters range. LTP must exceed LTD at MF-MVN [file pcbi.1006298.s002.pdf]

A

VOR Gain

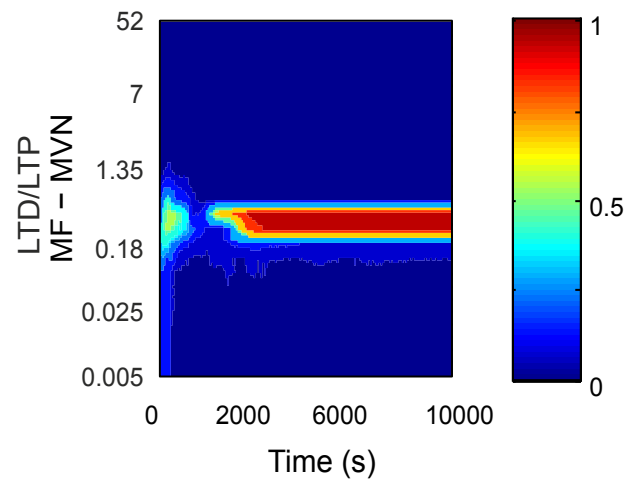

VOR Phase

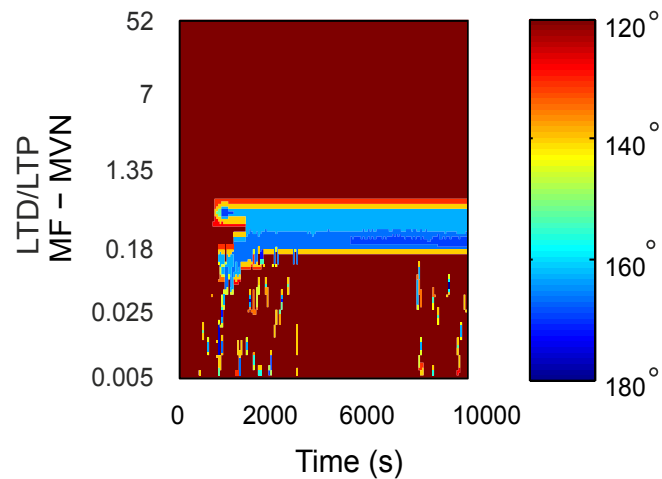

B

VOR  
Gain & Phase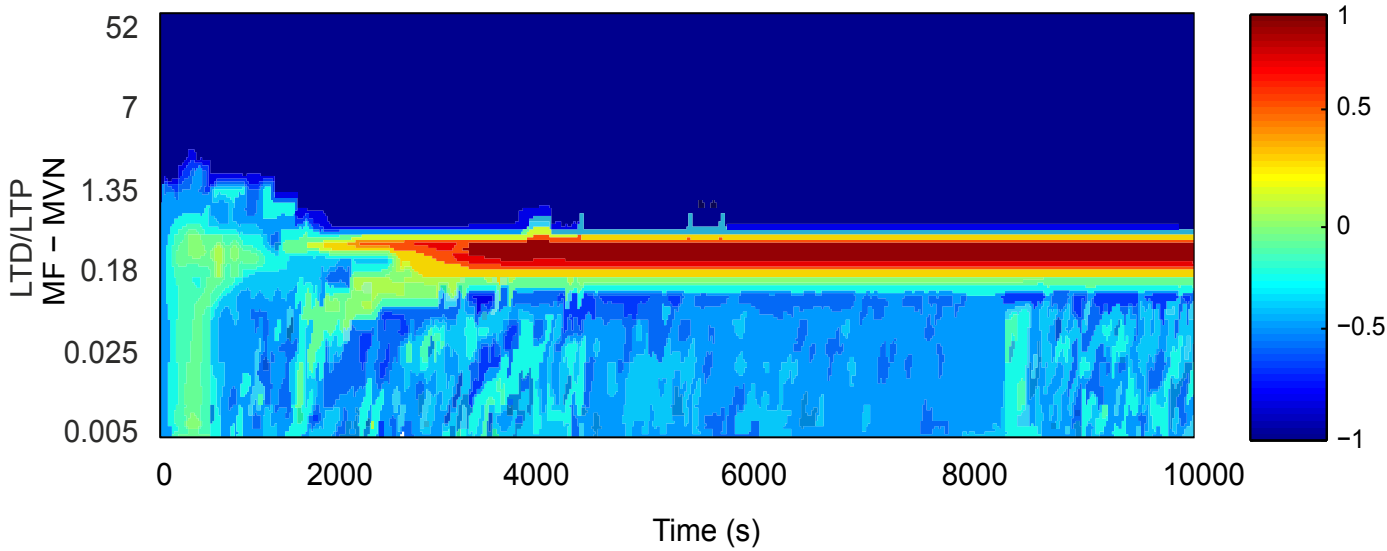

Supplement: S3 Fig — Whilst LTD/LTP balance was fixed at PF-PC synapses, we modified the LTD/LTP balance at MF-MVN synapses by systematically varying the ratio by 1.5N where –11 ≤ N ≤ 12 during a 10000 s simulation. (A) Final VOR gain and phase plotted as a function of the tested LTD/LTP range across time. (B) Combined VOR gain and phase (normalised) over time. A proper balance between LTD and LTP (ratio of approximately 0.4) makes the cerebellum perform optimally after 750 sec. (PDF) [file pcbi.1006298.s003.pdf]

A

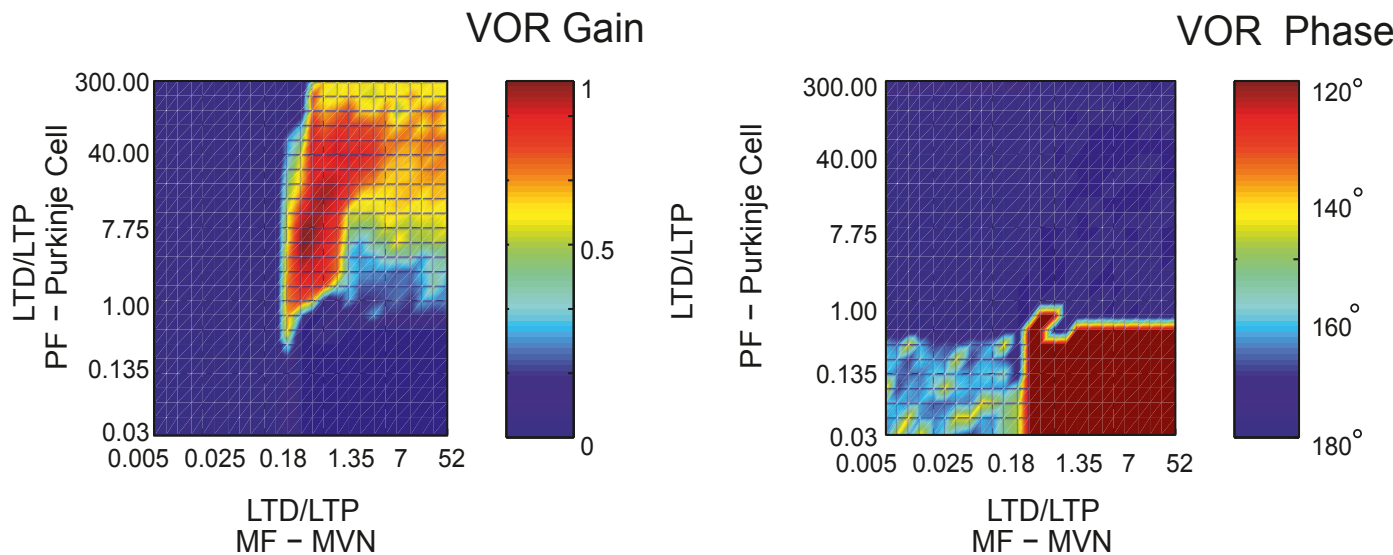

B

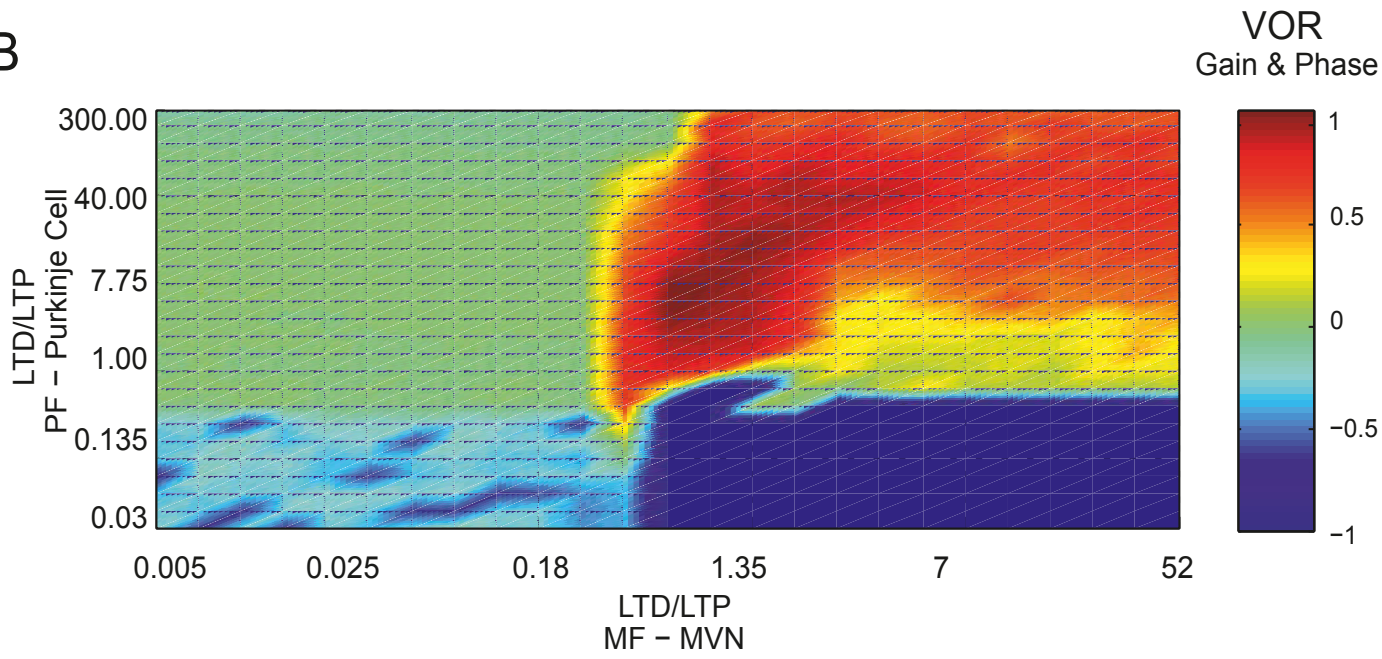

Supplement: S4 Fig — Similar to S2 Fig, the parameters regulating the LTD/LTP ratio were exhaustively tested whilst the cerebellar model without Purkinje complex spiking underwent h-VOR learning during a 10000 s simulation. (A) Final VOR gain and phase plotted over the LTD/LTP range of tested values. (B) Combined VOR gain and phase (normalised) as a function of the LTD/LTP ratio. LTD/LTP at both PF-Purkinje cell synapses is well balanced for N values ranged between [–1, 7]. Thus, the absence of bursting and pause dynamics leads to a wider range values for the LTD/LTP balance. (PDF) [file pcbi.1006298.s004.pdf]

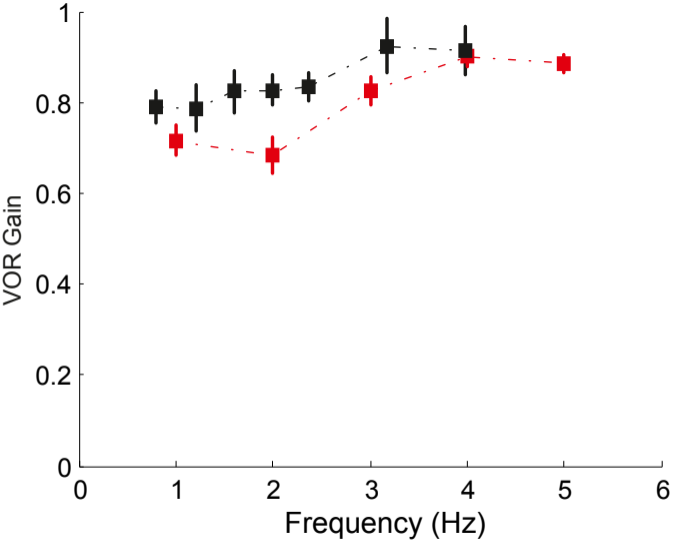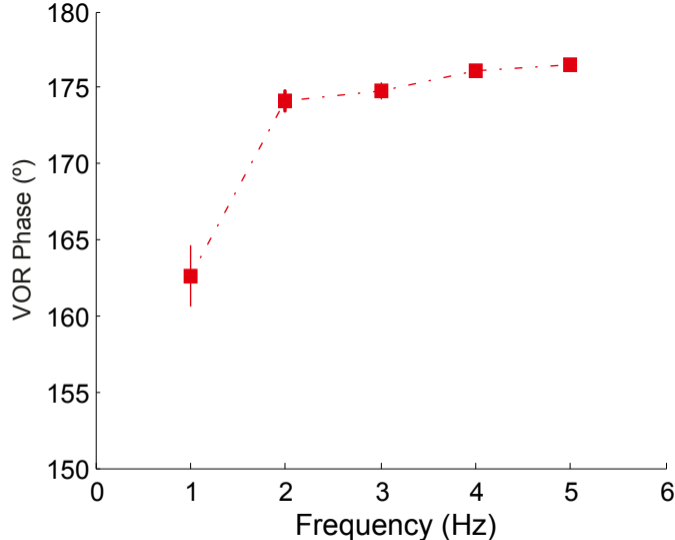

■ Human data ■ Model data

Supplement: S5 Fig — Average VOR gain/phase calculated by taken gain values each 400 s over the last 4000 s of the VOR adaptation process (10000 s) over a range of frequencies within the natural head rotation range [0.05-5Hz]. Consistently with the known frequency spectrum of the vestibular system [163], VOR gain remained relatively stable across the tested frequencies. (PDF) [file pcbi.1006298.s005.pdf]

A

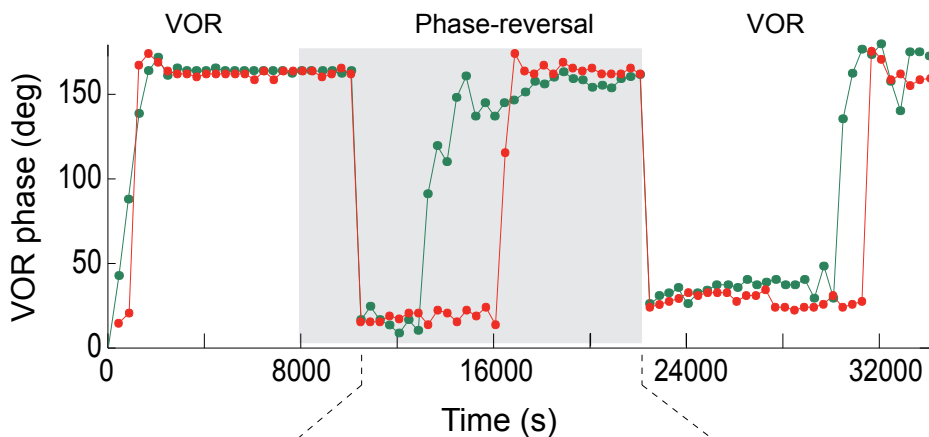

B

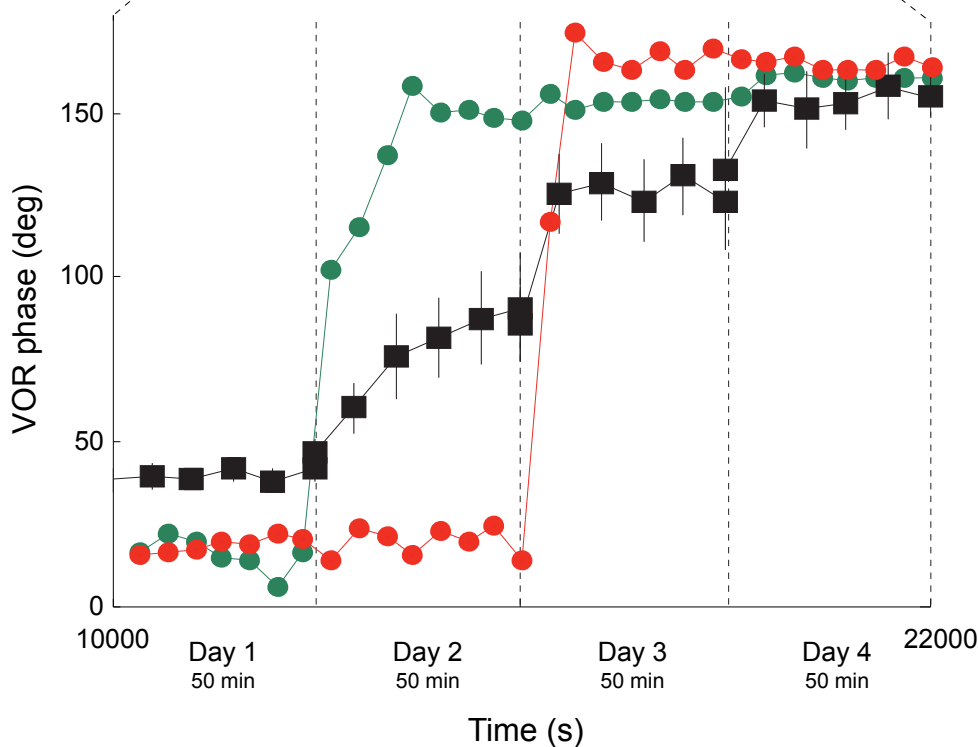

■ Wild mice data ● CS Purkinje Cell ● no-CS Purkinje Cell

Supplement: S6 Fig — Time course of the VOR phase. (A) VOR phase adaptation with (red curve) and without (green curve) Purkinje spike burst-pause dynamics. (B) Focus is on the phase-reversal period and comparison with experimental data [2]. (PDF) [file pcbi.1006298.s006.pdf]

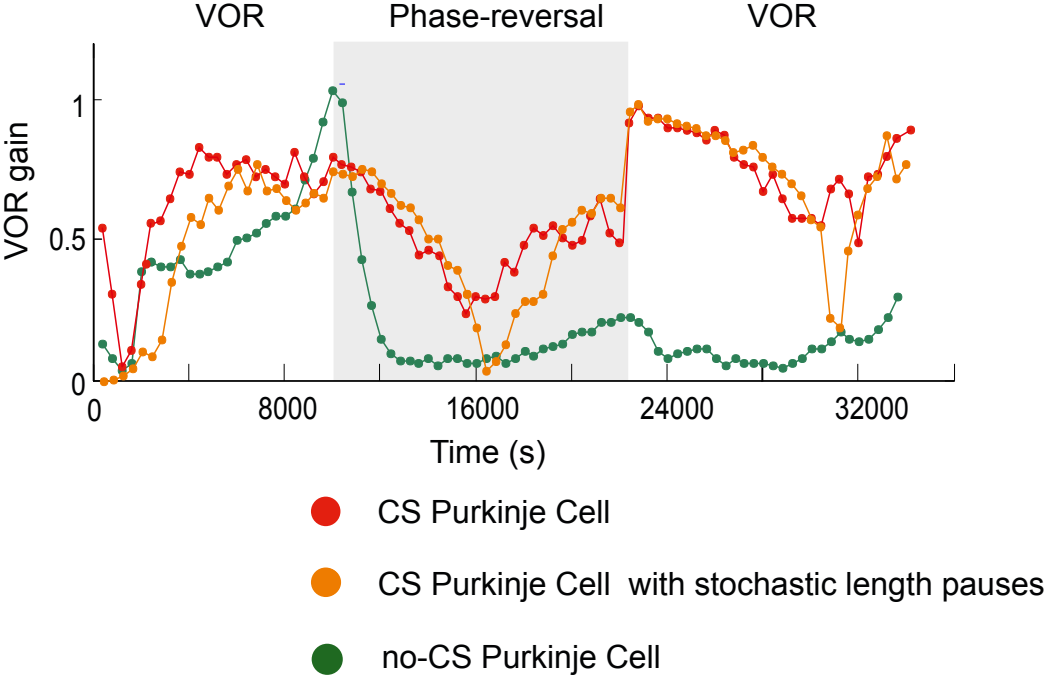

Supplement: S7 Fig — VOR gain adaptation mediated by the model with Purkinje spike burst-pause dynamics (orange and red curves, with stochastic vs. burst-dependent pause lengths, respectively; S1 Fig) and by the model without spike burst-pause dynamics (green curve). The simulated protocol is the same of Fig 5: VOR adaptation (first 10 000 s), phase-reversal learning (subsequent 12 000 s), and VOR restoration (remaining 12000 s). The presence of Purkinje spike burst-pause dynamics, regardless the relation between CF burst sizes and pause lengths (S1 Fig), improves the performance of cerebellar-dependent VOR adaptation. (PDF) [file pcbi.1006298.s007.pdf]

**A** CS Purkinje Cell

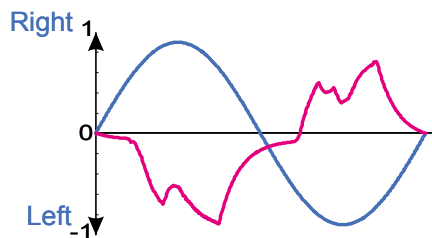

$t=10000$  s

500 ms

no-CS Purkinje Cell

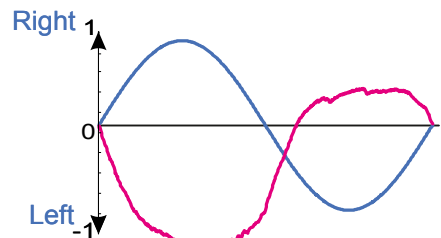

**B**

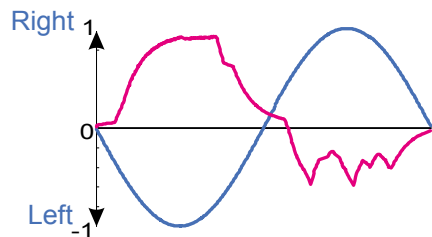

$t=22000$  s

500 ms

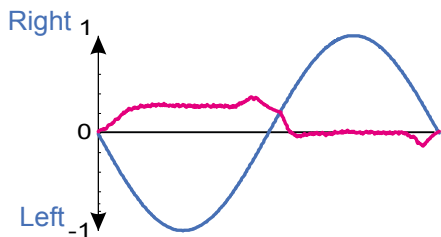

**C**

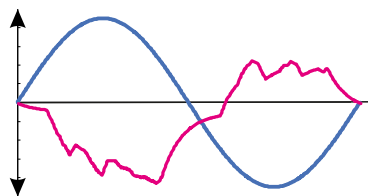

$t=32000$  s

500 ms

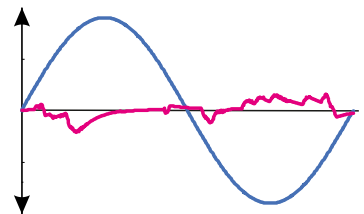

Supplement: S8 Fig — (A) Only the eye velocity movement corresponding to the sparser and more selective distribution of MF-MVN synaptic weights is able to counteract the head velocity movement in counter phase (B), as phase-reversal learning is achieved (C). (PDF) [file pcbi.1006298.s008.pdf]

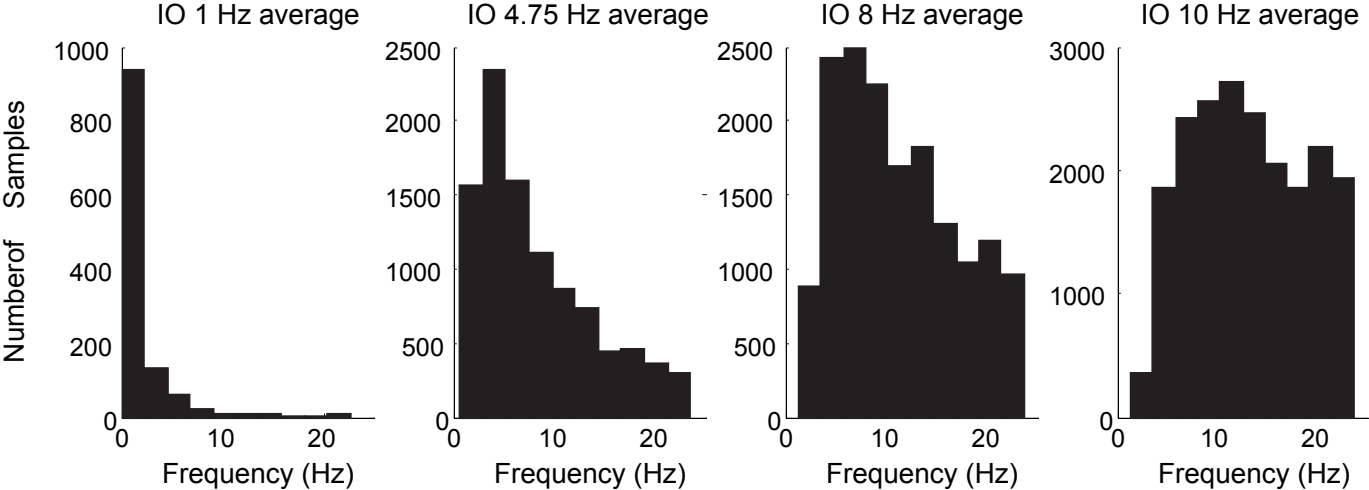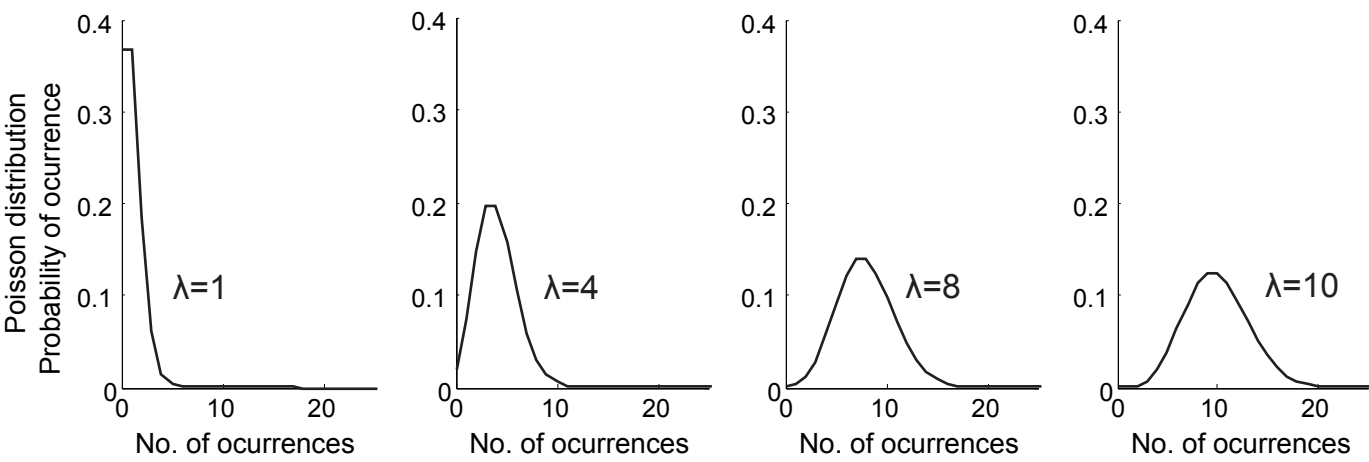

Supplement: S9 Fig — In the model, CF responses follow a probabilistic Poisson process. Given the normalised error signal ε(t) obtained from the retina slip and a random number η(t) between 0 and 1, the model CF fires a spike if ε(t)>η(t); otherwise, it remains silent[84] A single spike is then able to report timed information regarding the instantaneous error. Furthermore, the probabilistic spike sampling of the error ensures that the entire error region is accurately represented over trials with a constrained CF activity below 10 spikes per second, per fibre (CF activated between 1–10 Hz). Hence, the error evolution is accurately sampled even at a low frequency [148, 150]. This firing behaviour is consistent to those observed in neurophysiological recordings [149]. (PDF) [file pcbi.1006298.s009.pdf]
